# The role of transcription factor StBEL11 in carbon allocation and tuberization in cultivated potato differs from that known for the model Andean genotype

**DOI:** 10.1093/jxb/eraf551

**Published:** 2025-12-24

**Authors:** Andrea Zounková, Magdaléna Dybová, Helena Lipavská, Tomáš Mašek, Lukáš Fischer, Petra Mašková

**Affiliations:** Department of Experimental Plant Biology, Faculty of Science, Charles University, Viničná 5, Prague 2 128 00, Czech Republic; Department of Experimental Plant Biology, Faculty of Science, Charles University, Viničná 5, Prague 2 128 00, Czech Republic; Department of Experimental Plant Biology, Faculty of Science, Charles University, Viničná 5, Prague 2 128 00, Czech Republic; Department of Genetics and Microbiology, Faculty of Science, Charles University, Viničná 5, Prague 2 128 00, Czech Republic; Department of Experimental Plant Biology, Faculty of Science, Charles University, Viničná 5, Prague 2 128 00, Czech Republic; Department of Experimental Plant Biology, Faculty of Science, Charles University, Viničná 5, Prague 2 128 00, Czech Republic; University of California, Davis, USA

**Keywords:** BEL1-like transcription factor, biomass allocation, carbohydrates, phloem exudates, photosynthesis, RNAi, *Solanum tuberosum* ssp. *tuberosum*, source–sink interactions, StBEL11, tuberization

## Abstract

The transcription factor StBEL11 is known as a negative regulator of tuberization in the Andean potato (*Solanum tuberosum* ssp. *andigena*). This study focuses on its role in the common potato ssp. *tuberosum* by analysing transgenic lines of cv. Kamýk with down-regulated *StBEL11* expression. As expected, these lines showed enhanced tuber induction on nodal segments cultivated *in vitro* on high-sucrose medium. However, intact plants *ex vitro* exhibited significantly delayed tuberization and decreased yield, which surprisingly applied especially for lines with the most enhanced *in vitro* tuberization. Interestingly, reduced leaf *StBEL11* expression was accompanied by decreased leaf expression of the positive tuberization regulator *StBEL5*, but not of *SP6A* tuberigen. The transgenics showed increased photosynthetic activity, which was not, however, associated with non-structural carbohydrate accumulation. Unexpectedly, *StBEL11* down-regulation altered assimilate allocation in favour of roots over shoots and stolons before onset of tuberization and, in lines with the strongest phenotype, also during the early stage of tuberization, which probably explains their reduced tuber yields. The unexpected differences in tuber induction and yield between *andigena* and *tuberosum* lines with down-regulated *StBEL11* indicate changes in the strength/hierarchy of individual components of the tuberization regulatory network and point to the impossibility of easy knowledge transfer from Andean to cultivated potato.

## Teaser

RNAi-induced down-regulation of BEL11, a proposed negative regulator of tuberization in Andean potato, resulted in markedly different effects on tuberization, photosynthetic characteristics, and biomass allocation in cultivated potato.

## Introduction

The potato (*Solanum tuberosum*) is the fourth most important crop worldwide, playing a crucial role in the human diet due to its rich nutrient content. Potato tubers, the underground storage organs, provide significant amounts of carbohydrates, dietary fibre, vitamins, proteins, and minerals (Zaheer and Akhtar, 2016). Potatoes have long been a staple food in many countries, and their cultivation is expanding globally due to their adaptability to different conditions and their important contribution to food security. Significant increases in production have recently occurred in China, India, and various African countries (Devaux *et al*., 2020). To meet future demands, progress in potato cultivation and yields through knowledge-based breeding is essential. Tuberization—tuber formation from the tip of below-ground stems, stolons—is regulated by a complex interplay of genetic, metabolic, and environmental factors (reviewed, for example, by Zierer *et al*., 2021). While an environmental effect on tuberization (e.g. photoperiod, temperature, and nitrogen availability) has long been recognized, the molecular mechanisms underlying tuber formation have only recently been explored. Understanding the regulation of tuberization is essential for several reasons: breeding of varieties with enhanced yield; resistance to pests and diseases; and improved adaptability to changing environmental conditions, which is crucial for ensuring food security in the face of climate change. Indeed, climate scenarios predict a global decline in potato yields of up to 26% by 2085 (Raymundo *et al*., 2018), and in Europe even up to 84.6% (Gouerou *et al*., 2025). To date, molecular research, predominantly using the model genotype of ssp. *andigena*, has provided basic insights into key regulatory biomolecules (proteins, RNAs, and phytohormones) driving tuberization (e.g. Kondhare *et al*., 2020). Interestingly, tuber initiation shares some features with flowering onset, particularly in terms of signal production and its mobility (Abelenda *et al*., 2014). Navarro *et al*. (2011) demonstrated that SELF-PRUNING 6A (SP6A), a homologue of the florigen FLOWERING LOCUS T, responds to environmental cues and acts as a long-distance tuberization signal. Under favourable conditions, SP6A protein is produced in leaves and transported via the phloem to stolons, where it interacts with FLOWERING LOCUS D-like and 14-3-3 proteins to form a tuberigen activation complex; this regulates the expression of target genes (Teo *et al*., 2017; Wang *et al*., 2023). Notably, the mobile tuberigenic signal is more complex and comprises other mobile signals including transcripts for the BEL1-LIKE (BEL) and KNOTTED1-like HOMEOBOX (KNOX) transcription factors, specifically *BEL5*, *BEL11*, and *BEL29* mRNAs from the BEL family, and *POTATO HOMEOBOX 1* (*POTH1*) mRNA from the KNOX family (Banerjee *et al*., 2006; Mahajan *et al*., 2012; Ghate *et al*., 2017; recently reviewed by Zounkova *et al*., 2024). While BEL5 and POTH1 were identified and characterized earlier, the functions of BEL11 and BEL29 remain much less explored. In various potato genotypes, overexpression of *BEL5* as well as of *POTH1* positively correlates with enhanced tuber yields (Chen *et al*., 2003; Rosin *et al*., 2003; Cho *et al*., 2015; Sharma *et al*., 2016). Further, BEL5 and POTH1 were shown to form a heterodimer that regulates the expression of target genes by binding to their promoter sequence containing tandem TTGAC core motifs (Chen *et al*., 2003, 2004; Lin *et al*., 2013; Sharma *et al*., 2016). These target genes include, among others, *SP6A*, thus amplifying the tuberigenic signal (Sharma *et al*., 2016). Other target genes encode proteins involved, for example, in metabolism regulation, phytohormone transport and signalling, cell cycle control, and carbohydrate transport (Chen *et al*., 2003, 2004; Lin *et al*., 2013; Sharma *et al*., 2016). In contrast, BEL11 and BEL29 were proposed to be negative regulators of tuberization, counteracting the effect of BEL5. However, it has been only shown in the strictly short-day (SD) tuberizing potato genotype of ssp. *andigena* (Ghate *et al*., 2017). Molecular mechanisms underlying BEL11 and BEL29 action remain unclear as the knowledge so far only comes from a single study. BEL11 and BEL29 also possess putative DNA-binding domains, but their ability to enter the nucleus, bind to target gene promoters, and therefore function as transcriptional regulators has yet to be validated. Since all three mentioned BEL proteins can interact with POTH1, BEL11 and BEL29 might negatively affect BEL5 function by competing for the POTH1 interaction partner (Ghate *et al*., 2017).

Considering the extreme genomic diversity of potato, shaped by both wild hybridization and domestication (Hardigan *et al*., 2017), we wondered whether the negative effect of BEL11 on tuberization, previously demonstrated in the potato landrace of ssp. *andigena*, is conserved in cultivated potato of ssp. *tuberosum*. Although the two subspecies differ in several traits, including environmental adaptations [e.g. long-day (LD) tuberization in ssp. *tuberosum*] and patterns of biomass allocation, the underlying differences in the functioning of regulatory pathways remain largely unknown. To investigate this, we generated transgenic lines of ssp. *tuberosum* cv. Kamýk that expressed part of the *BEL11* gene in the antisense orientation to down-regulate expression of the native *BEL11* gene by RNA interference, hereinafter called BEL11 RNAi lines. To thoroughly characterize these lines, we evaluated tuberization under a set of cultivation regimes, quantified selected transcript levels, carbohydrate production and distribution, growth characteristics, and biomass allocation. Interestingly, the effects of *BEL11* down-regulation differed from those described in ssp. *andigena*, as weakening this hypothetical tuberization inhibitor did not lead to the expected enhancement of tuberization but, surprisingly, resulted in significant suppression under natural-like conditions. These differences were likely to be related to the observed positive effects of *BEL11* down-regulation on photosynthetic activity and to altered biomass allocation within the plant body.

## Materials and methods

### Preparation of potato transgenic lines

To generate BEL11 RNAi suppression lines, we employed the potato (*S. tuberosum*) cultivar Kamýk that is a traditional, intermediate to late maturing, easy transformable genotype sourced from the breeding station Sativa Keřkov, a.s., the Czech Republic. We utilized a construct comprising a 401 bp fragment containing a unique region of the *BEL11* coding sequence (CDS), along with a small portion of the 5'-untranslated region (UTR), both in antisense orientation, under the control of the cauliflower mosaic virus (CaMV) *35S* promoter, cloned into the binary vector pCB201 (Ghate *et al*., 2017), kindly provided by Professor D. Hannapel from Iowa State University, USA. The transformation process was mediated by *Agrobacterium tumefaciens* strain C58C1. The stably transformed plant lines were obtained through the transformation of leaf explants and *de novo* regeneration, employing a modified protocol of Dietze *et al*. (1995). Stable transformation of the regenerated plants was verified by PCR using transgene-specific primers (Supplementary Table S1). DNA isolation was carried out using a modified rapid DNA isolation protocol based on Edwards *et al*. (1991) (with an additional step of chloroform purification and DNA dissolution in 100 μl of 2 mM Tris buffer, pH 8.5). The positively genotyped lines were then tested for the level of *BEL11* transcript by reverse transcription– quantitative PCR (RT–qPCR; for details, see ‘Determination of transcript levels’) and four lines with a significantly decreased *BEL11* transcript level were selected for further experiments.

### Plant cultivation

#### In vitro

Plant maintenance and multiplication under *in vitro* conditions were carried out using single-node cuttings taken from 4-week-old *in vitro* plants cultivated on Murashige and Skoog (MS; Murashige and Skoog, 1962) medium either mixotrophically (3% sucrose; w/v) or photoautotrophically (without sucrose, gas-permeable lid; for details, see Ševčíková *et al*. (2019), under an LD photoperiod (16/8 h), photosynthetically active radiation (PAR) of 150 μmol m^–2^ s^–1^, and warm white fluorescent tube light, 22±2 °C.

#### *Ex vitro* in hydroponics

For hydroponic cultivation, we used a Low-density growing system (Araponics, Liège, Belgium). The holders in the lid were modified to enable ‘underground’ shoot (stolon) growth of potato plants after *ex vitro* transfer. Roots and a part of the shoot which arose *in vitro* (always comprising the same number of nodes) of mixotrophically pre-cultivated plants were placed below the lid. For each hydroponic system, 12 plants were used and cultivated in 1.5 litres of modified 1/4 Hoagland solution (Hoagland and Arnon, 1938), PAR 120 μmol m^–2^ s^–1^, white LED supplemented with far red LED light, 22±2 °C.

#### *Ex vitro* and tuber-derived plants in soil

*In vitro* mixotrophically pre-cultivated plants (4 weeks) were used for *ex vitro* experiments performed under an LD photoperiod (16/8 h), PAR 310 μmol m^–2^ s^–1^, a combination of warm white and cool white fluorescent tube light, 22±2 °C (cultivation chamber), or an LD-adjusted photoperiod (16/8 h), 18±2 °C (greenhouse). Alternatively, post-dormant tubers of comparable size (weight ∼10–15 g) were planted in a greenhouse under the same conditions. Plants were cultivated in either 7 litre pots (greenhouse tuberization) or 1 litre pots (other experiments); substrate consisted of common garden substrate, compost soil, sand, and perlite (6:2:1:2).

### Tuberization experiments

#### In vitro

Single-node segments with leaf blades removed were excised from mixotrophically *in vitro* grown plants and cultivated for 5 weeks (35 days after transfer, DAT) on tuber-inducing medium [TIM: MS with 0.2 mg l^−1^ 6-benzylaminopurine (BAP) and 8% sucrose (w/v)] under constant darkness, 22±2 °C. The number of micro-tubers was evaluated weekly (7, 14, 21, 28, and 35 DAT); all other parameters were determined at 35 DAT.

#### *Ex vitro* in hydroponics

*In vitro* mixotrophically pre-cultivated (4 weeks) plants were used and cultivated under an SD photoperiod (8/16 h). The appearance and number of tubers were recorded weekly (7, 14, 21, 28, and 35 DAT); all other parameters were determined at 35 DAT.

#### *Ex vitro* and tuber-derived plants grown in soil

For tuberization experiments, plants were cultivated in the cultivation chamber and in the greenhouse. All parameters were evaluated at the end of the vegetation period (130 DAT).

### Growth characteristics

Growth characteristics were evaluated in non-induced plants, as well as in plants with initiated tuberization. Non-induced plants were cultivated either photoautotrophically *in vitro* or *ex vitro* in hydroponics (after 3 weeks of mixotrophic pre-cultivation *in vitro*) under an LD photoperiod (16/8 h); growth characteristics were evaluated 21 DAT. Plants destined for tuber induction were cultivated *ex vitro* in hydroponics (after 4 weeks of mixotrophic pre-cultivation *in vitro*) under an SD photoperiod (8/16 h); growth characteristics were evaluated 28 DAT (pre-tuberization stage) and 41 DAT (early tuberization stage).

### Photosynthetic characteristics

Photosynthetic characteristics were determined 21 DAT in *ex vitro* soil-grown plants cultivated in a cultivation chamber.

The net photosynthetic rates of intact fully developed leaves (6 cm^2^ measured) were determined gasometrically using a LI-6400 portable gas analyser (LI-COR, Lincoln, NE, USA) at 21 °C, 500 µmol CO_2_ mol^−1^. The light exposure protocol consisted of a 10 min dark adaptation phase and subsequent irradiations in the following sequence: 100, 300, 500, 700, and 900 µmol photons m^−2^ s^−1^ (5 min interval each). The measured values were collected at 1 min intervals.

The fast fluorescence kinetics were determined on dark-adapted intact leaves using a pocket fluorometer FluorPen 2 (PSI, Photon System Instrument, Czech Republic). The measurements were repeated at two positions on the leaf blade. The maximum quantum yield of primary PSII photochemistry was calculated as (*F*_m_–*F*_o_)/*F*_m_, where *F*_m_ is the maximum value of fluorescence under saturating irradiance and *F*_o_ is the initial fluorescence value.

For determination of photosynthetic pigment contents, cut-outs from fully developed leaves (81 mm^2^) were plunged into 4 ml of *N*,*N*-dimethylformamide and incubated in darkness at 4 °C for 1 week to extract the photosynthetic pigments. The pigment contents (chl *a*, chl *b*, and total carotenoids) were determined spectrophotometrically (Evolution 201, program Thermo insight) by measuring absorbance at 480, 647, 664, and 750 nm, and calculated according to Wellburn (1994).

### Determination of carbohydrate content

Tissue carbohydrate contents were determined in soil-grown *ex vitro* plants (cultivation chamber, LD photoperiod, 16/8 h) and in hydroponically cultivated plants under both LD (16/8 h) and SD (8/16 h) photoperiods.

Samples (50–150 mg FW) of the leaves, stolons/tubers, and roots collected 21 DAT (LD), and 28 and 41 DAT (SD) were immediately frozen in liquid nitrogen, then freeze-dried and boiled in 80% methanol (v/v) at 75 °C for 15 min. The solvent was then vacuum-evaporated and the residue resuspended in Milli-Q ultrapure water (Millipore, Bedford, MA, USA) before being purified by centrifugation and filtration. The content of non-structural soluble carbohydrates was determined using HPLC as described in Kofroňová *et al*. (2020). The starch in the pellets after extraction of the soluble carbohydrates was hydrolysed in 0.5 ml of 0.1 M Na-acetate buffer (pH 4.5) by autoclaving and then enzymatically cleaved with α-amylase (Sigma, 30 U per sample) and amyloglucosidase (Sigma, 60 U per sample) at 40 °C overnight, the solvent was then vacuum-evaporated and the pellets processed similarly to soluble saccharide samples and measured by HPLC (Kofroňová *et al*., 2020). Starch content was expressed as the amount of glucose per DW unit. The data were evaluated using Clarity 7.2 software (DataApex).

### Determination of transcript levels

To evaluate the extent of RNAi, the levels of *BEL11* mRNA (target gene) were determined in leaves of 4-week-old *in vitro* plants. Subsequently, more detailed quantification of *BEL11* mRNA and other selected transcripts (*BEL5*, *BEL29*, *POTH1*, and *SP6A*) was performed in the source and sink organs (leaves, stolons, and roots) of *ex vitro* soil-cultivated plants, grown in the cultivation chamber. Moreover, *BEL11* transcript levels were also examined in phloem exudates (see ‘Phloem exudation’). To compare with later developmental stage, transcript analysis was also performed in leaves and young tubers of SD-induced plants grown in hydroponics. Total RNA was isolated from plant material (except tubers) using the TRI reagent isolation protocol (Sigma Aldrich). For tuber samples (rich in starch), we employed the RNeasy PowerPlant Kit (QIAGEN). The root samples were further purified using the RNA Clean and Concentrator kit (Zymo Reserach, USA) according to the instructions of the manufacturer. RNA integrity was checked by RNA denaturing agarose gel electrophoresis (Masek *et al*., 2005). A 8 µg aliquot of total RNA from each sample was reverse-transcribed using 200 U of RevertAid™ reverse transcriptase (Thermo Scientific) and 0.5 μg of oligo(dT) in a reaction volume of 40 µl. cDNA synthesis was performed at 37 °C for 5 min, followed by incubation at 42 °C for 1 h and subsequent inactivation at 70 °C for 10 min. The levels of selected transcripts were determined by RT–qPCR in LightCycler 480 (Roche) using LightCycler 480 SYBR Green I Master (Roche) in 10 μl reactions (three technical replicates for each template dilution). Each reaction contained 2 µl of cDNA and 500 nM gene-specific primers (a list of the primers used is provided in Supplementary Table S1). To avoid possible genomic DNA amplification, we designed primers spanning over exon junctions whenever possible or annealing to distantly positioned exons. We also included reverse transcriptase-free reactions as negative controls. Our data published earlier (Ševčíková *et al*., 2017) showed that transcripts of elongation factor 1α-like as well as polyubiquitin exhibit highly stable expression across organs and conditions in cultivated potato, and thus were shown to be suitable as reference transcripts for data normalization of qPCR. Based on this, we utilized *polyubiquitin* (*UBI*) mRNA as a reference in this study. The amplification program consisted of: initial template denaturation, 95 °C 5 min; then 45 cycles (denaturation, 95 °C 10 s, primer annealing, 58 °C 15 s, and extension, 72 °C 10 s), followed by melting curve analysis; 97 °C 10 s, 65 °C 1 min, and by slow warming to 97 °C (ramp rate: 0.11 °C s^–1^, five signal reads per °C). The LightCycler 480 software (version 1.5) was applied to calculate relative copy numbers of the analysed mRNAs in relative quantification mode with standard curves in the run. An overview of RT–qPCR assay parameters for the wild type (WT) under both cultivation conditions is provided in Supplementary Table S2. Additionally, the specificity of amplification with individual primer pairs was analysed by melting curve genotyping and DNA electrophoresis of the respective PCR products.

### Phloem exudation

Phloem exudates were collected 21 DAT using *ex vitro* soil-cultivated plants (greenhouse) and 21, 28, and 41 DAT using tuberization-induced plants in hydroponics under SD. The protocol was based on Tetyuk *et al*. (2013). Whole shoots were cut out using a razor blade near the soil surface and the cut area was immersed in MiliQ water. Another transversal section removed air bubbles from transporting tissues. Shoots were then placed in high air humidity conditions and pre-incubated in 20 mM K_2_EDTA for 1 h to prevent plugging of sieve elements. For saccharide determination, phloem exudates were collected in MiliQ water for another 1 h. Samples were then dried using a vacuum-evaporator, re-dissolved in 20 µl of MiliQ water, and saccharide contents were measured using HPLC as described above. The rate of exudation was expressed as the content of saccharides collected per hour per leaf area unit. For determination of *BEL11* transcript phloem mobility, after K_2_EDTA pre-treatment, phloem exudates were collected for 1 h in a 1 ml MiliQ water solution containing 80 U ml^−1^ RNase inhibitor (RiboLock, ThermoFisher), 15% (v/v) protease inhibitor (Complete, Mini, EDTA-free, Roche, 7× stock, one tablet dissolved in a final volume of 10 ml), and 50 µg ml^−1^ linear polyacrylamide (GeneElute, Sigma). To minimize the effect of RNA losses during sample processing, the collected exudates were spiked with a 3 µl aliquot of TATAA Universal RNA Spike II spike transcript (TATAA Biocenter AB, Goteborg, Sweden) and then mixed with an equal volume of isopropanol for RNA overnight precipitation at −70 °C. Subsequently, samples were centrifuged, the pellet was dried and re-dissolved in RNase-free water, and RNA was isolated using 0.5 ml of TRI reagent and further processed as described above, including cDNA synthesis and RT–qPCR. The rate of exudation was expressed as the relative *BEL11* transcript to spike transcript level per plant per hour.

### Statistical analysis

Boxplot graphs were generated using JASP software (Version 0.18.3). Error bars in bar charts represent standard deviations. NCSS 9 statistical software (NCSS, LLC. Kaysville, UT, USA) was employed for data analysis. ANOVA was used for data meeting the criteria of normal distribution and homogeneity of variance. For data with only biological replicates, one-way ANOVA was employed. If both biological and technical replicates were involved, repeated measures ANOVA was utilized. In both experimental arrangements, RNAi plants were compared with the control (WT) using Dunnett’s two-sided test (versus control). For data not meeting the assumptions of normality and homogeneity of variance, or data with a low number of replicates, the Kruskal–Wallis Z test (Dunn’s test) was used. Statistical significance was determined at *P*≤0.001, *P*≤0.01, *P*≤0.05, and *P*≤0.1 levels, labelled as ***, **, *, and (*), respectively.

## Results

### Selection of BEL11 RNAi transgenic lines

**Fig. 1.**
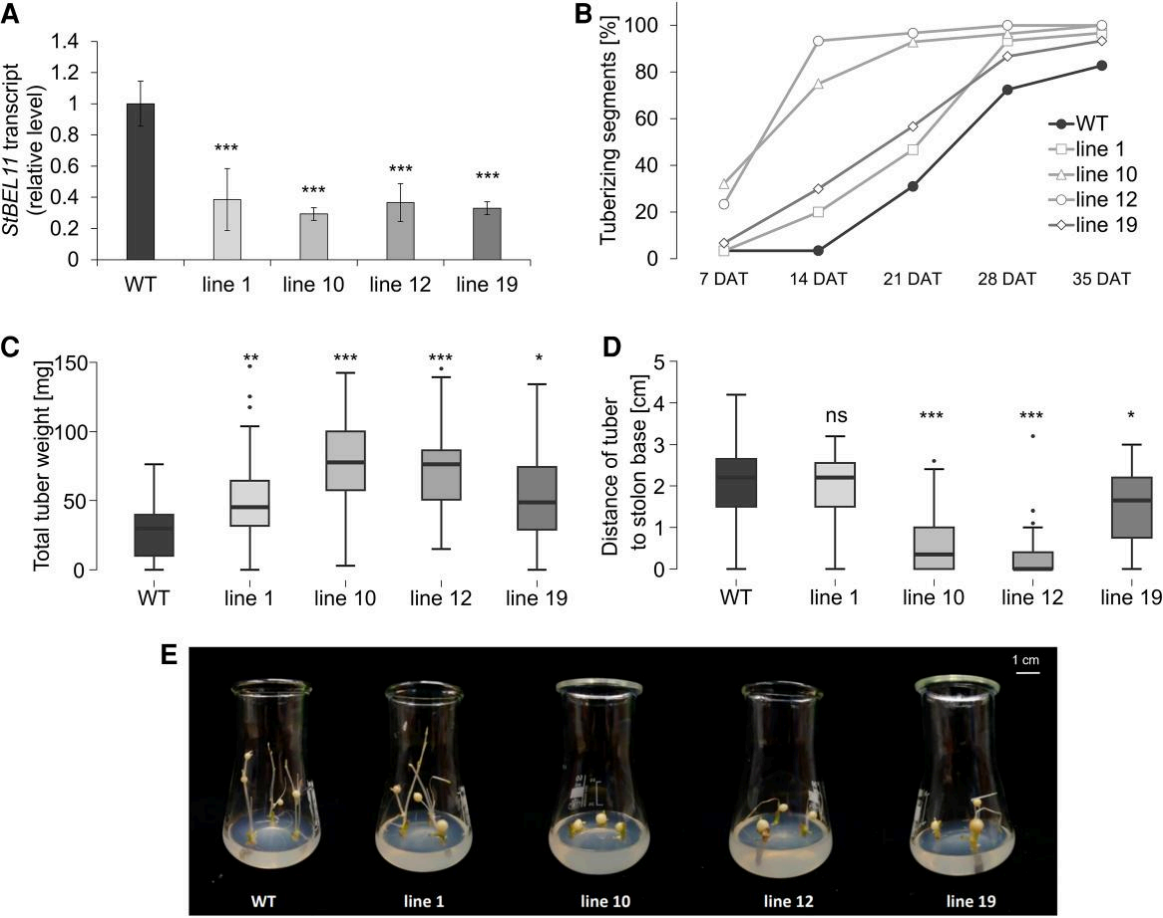
*BEL11* transcript level and tuberization *in vitro.* (A) Verification of decreased *BEL11* transcript level in lines 1, 10, 12, and 19 in 4-week-old *in vitro* cultivated plants under an LD photoperiod, normalized to the copy number of the reference transcript—*polyubiquitin*. The WT was set to 1. Three technical replicates were performed for each cDNA dilution (5× and 50×). (B) Timing of tuber onset. (C) Total tuber weight. (D) Average distance of tuber to stolon base. (E) Appearance of tuberizing stolons. For (B–E), node cuttings cultivated *in vitro* on TIM under darkness were evaluated 7, 14, 21, 28, and 35 DAT (B) and 35 DAT (C–E), respectively. The WT and four independent BEL11 RNAi lines: 1, 10, 12, and 19; for (A) *n*=3–11, for (B–D) *n*=28–30. For (A), error bars represent the standard deviations. Repeated measures ANOVA (A) or one-way ANOVA (C and D), Dunnett’s two-sided multiple-comparison test with control was used for statistical evaluation. Asterisks indicate statistically significant differences ***P≤0.001; **P≤0.01; *P≤0.05; ns, not significant..

Out of 10 stably transformed independent BEL11 RNAi lines that were screened for *BEL11* transcript levels (Supplementary Fig. S1A) we selected four lines (lines 1, 10, 12, and 19) with significantly reduced *BEL11* transcript levels (Fig. 1A) and used them for subsequent analyses.

### Tuberization *in vitro* started earlier in BEL11 RNAi transgenics

Tuber induction on node cuttings cultivated *in vitro* under tuber-inducing conditions was monitored weekly (7–35 DAT). Notably, all tested BEL11 RNAi lines (particularly lines 10 and 12) exhibited accelerated initiation of tuber formation, though tuberization efficiency at 35 DAT did not substantially differ from that of the WT (Fig. 1B). Nevertheless, all four transgenic lines had significantly higher total tuber weights at 35 DAT (Fig. 1C, E), particularly due to higher average tuber weight (Supplementary Fig. S1B, C). Moreover, all four lines displayed a higher proportion of tuber biomass (51–84%) to the sum of tuber and stolon biomass, in contrast to the WT (36%) (Supplementary Fig. S1D). In accordance with the earlier tuber onset, the transgenic lines (except line 1) showed a higher tendency for sessile (positioned close to the stolon base) tuber formation (particularly in lines 10 and 12) (Fig. 1D). Furthermore, all WT stolons exhibited branching, but a portion of transgenic segments (10, 56, 67, and 8% for lines 1, 10, 12, and 19, respectively) produced only non-branched stolons. Overall, these findings indicate a strengthened responsiveness of BEL11 RNAi nodal segments to tuber-inducing conditions *in vitro*, manifested by earlier tuber onset, increased biomass allocation to tubers, and suppressed stolon growth.

### BEL11 down-regulation had an inhibitory effect on tuberization in plants grown under natural-like conditions

**Fig. 2.**
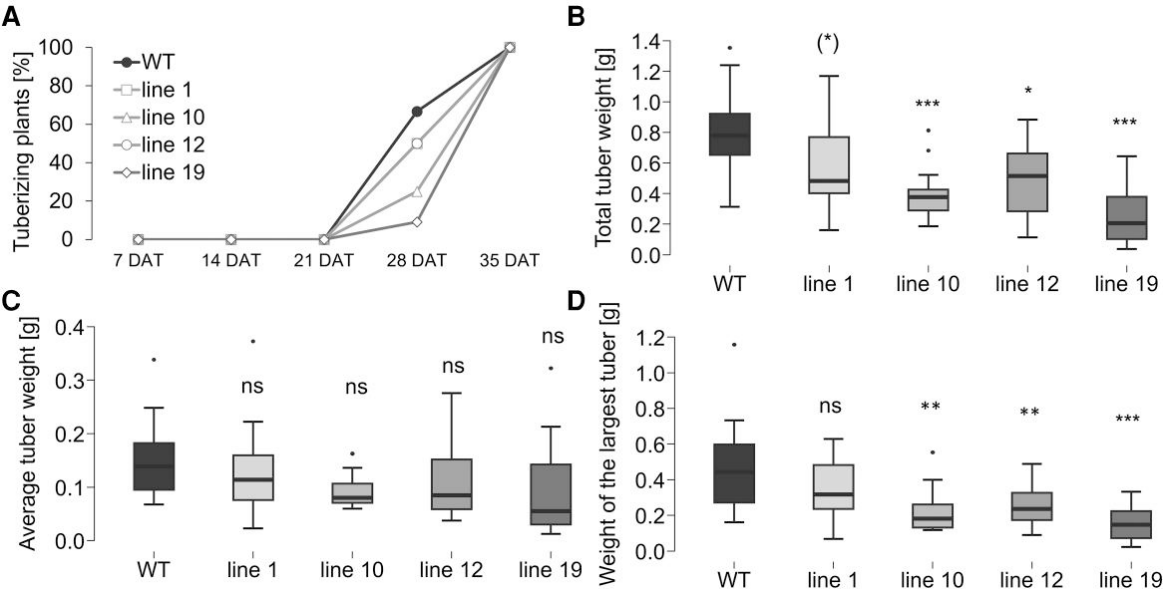
Tuberization *ex vitro* in hydroponics. (A) The timing of tuber onset, (B) total tuber weight, (C) average tuber weight, and (D) average weight of the largest tuber were calculated per plant cultivated in a hydroponic system under an SD photoperiod, evaluated 7, 14, 21, 28, and 35 DAT (A) and 35 DAT (B–D); WT and four independent BEL11 RNAi lines: 1, 10, 12, and 19; *n*=11–12. One-way ANOVA, Dunnett’s two-sided multiple-comparison test with control was used for statistical evaluation. Asterisks indicate statistically significant differences ***P≤0.001; **P≤0.01; *P≤0.05; (*)*P*≤0.1; ns, not significant.

**Fig. 3.**
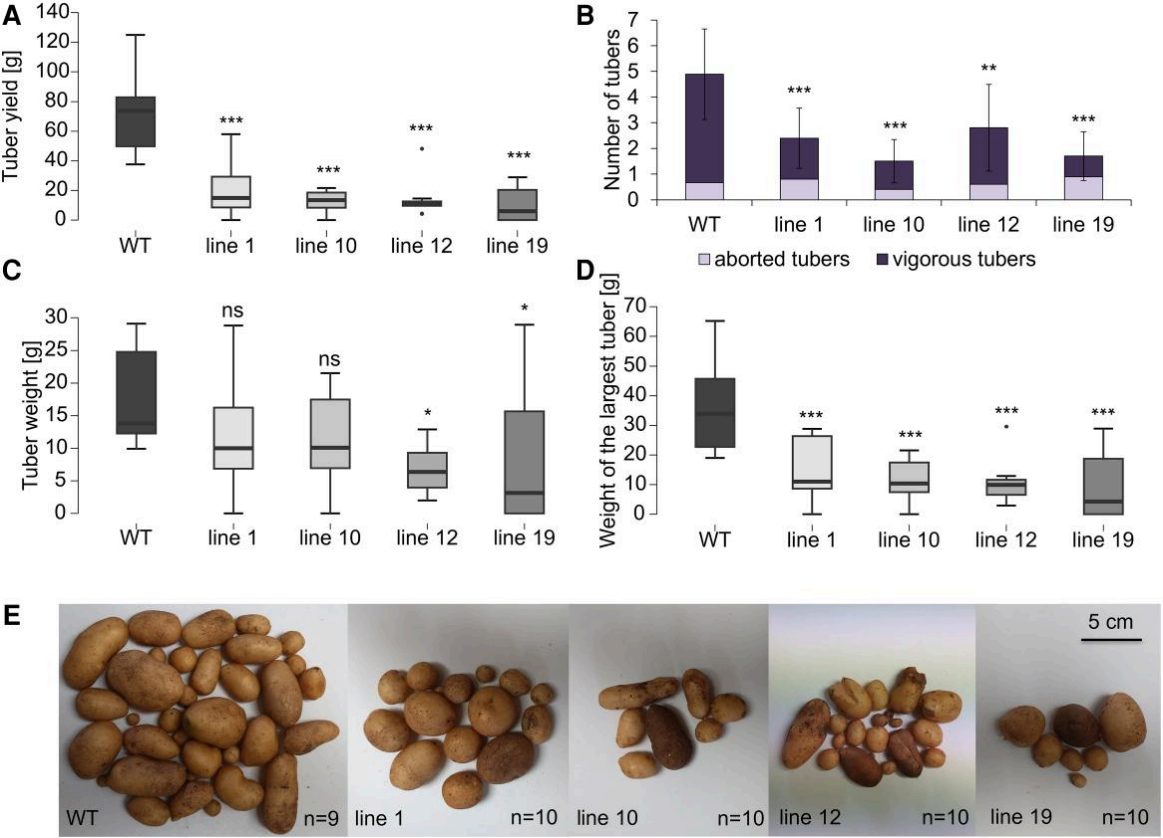
Tuberization of tuber-derived plants. (A) Average tuber yield, (B) average number of tubers, (C) average tuber weight (D), average weight of the largest tuber calculated per plant. (E) Photographs of tubers. (A, C, D, and E) involve only vigorous tubers of soil-cultivated plants under an LD photoperiod in the greenhouse, evaluated 130 DAT. WT and four independent BEL11 RNAi lines: 1, 10, 12, and 19; *n*=9–10. For (B), error bars represent the standard deviation. One-way ANOVA, Dunnett’s two-sided multiple-comparison test with control was used for statistical evaluation. Asterisks indicate statistically significant differences ***P≤0.001; **P≤0.01; *P≤0.05; ns, not significant.

Motivated by the results showing enhanced tuberization of all BEL11 RNAi lines *in vitro*, we continued with *ex vitro* experiments. In the hydroponic system, the first tubers appeared 28 DAT in 67% of WT plants, while in the BEL11 RNAi plants, tuber induction varied from 9% to 50% (Fig. 2A). Moreover, the WT showed a trend towards a higher number of swollen stolons—stage 2 according to Viola *et al*. (2001)—per plant (1.7) compared with the RNAi lines (0.9, 1.2, 0.8, and 0.2 for lines 1, 10, 12, and 19, respectively). Even at 35 DAT, the average number of tubers in RNAi lines did not exceed that of the WT (Supplementary Fig. S2). Total tuber weights per plant at 35 DAT were significantly reduced in RNAi lines (Fig. 2B), but the average tuber weights were comparable with that of the WT (Fig. 2C). Importantly, the weight of the largest tuber was higher in the WT (Fig. 2D). Overall, in the hydroponic system, tuber induction was delayed in the BEL11 RNAi lines compared with the WT, thus contrasting strongly with the induction under *in vitro* conditions. While both systems enabled simple monitoring of tuber induction, none of them represented natural conditions for potato cultivation. Therefore, we also tested the overall tuberization potential of *ex vitro* plants cultivated in soil (both cultivation chamber and greenhouse) to determine total tuber yield at the end of the vegetative season (130 DAT). For lines 1 and 19, the overall tuber yields, average tuber weights, and the weights of the largest tubers were comparable with those of the WT; however, these parameters were significantly lower in lines 10 and 12 (Supplementary Figs S3, S4), which in turn exhibited the highest tuber yields *in vitro* (Fig. 1). Although the phenotype of individual transgenic lines was more variable than in previous experiments, greenhouse results matched those from the cultivation chamber well, confirming stable and reproducible tuberization phenotypes of *ex vitro* soil-grown plants (Supplementary Fig. S3). Strikingly, the above-described phenotype of *ex vitro* grown plants was even stronger in tuber-derived plants, with dramatically reduced tuber yields of all RNAi lines to only 15–32% of the WT (Fig. 3A, E). This reduction was linked to a significantly lower number of tubers per plant compared with the WT (Fig. 3B). Although we found some aborted tubers (tubers degraded early after initiation of tuber filling) in each genotype, their portion remained consistent across genotypes. Average tuber weights, although highly variable, tended to be lower in transgenics (Fig. 3C) while the weights of the largest tuber significantly decreased in all RNAi lines (Fig. 3D). Altogether, these data further support the observed phenotype of suppressed tuberization in BEL11 RNAi lines *ex vitro*.

### BEL11 down-regulation led to unexpected changes in the expression pattern of tuberization-associated genes

**Fig. 4.**
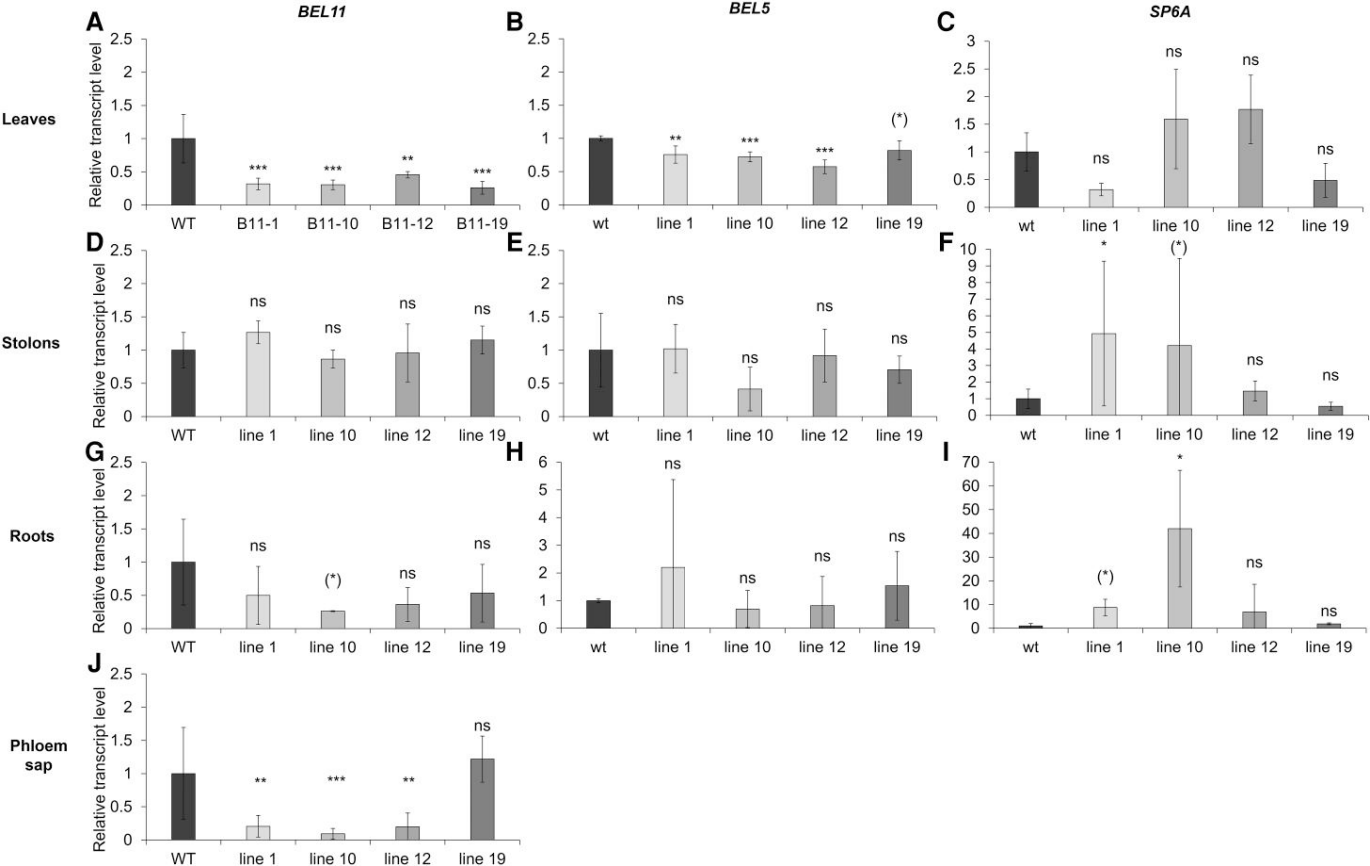
Selected transcripts levels in individual organs and phloem sap, (A, D, G, and J) *BEL11*, (B, E, and H) *BEL5*, (C, F, and I) *SP6A*. RNA was isolated from *ex vitro* soil-cultivated plants (cultivation chamber for A–I; greenhouse for J) under an LD photoperiod and evaluated 21 DAT. WT and four independent transgenic BEL11 RNAi lines: 1, 10, 12, and 19; normalized to (A–I) the copy number of the reference transcript—*polyubiquitin*, and (J) the external spike mRNA. Three technical replicates were performed for each cDNA dilution (5× and 50×). The WT level was set to 1 (mean Δ Ct values for the WT are provided in Supplementary Table S2). *n*=3–6; error bars represent the standard deviations. Repeated measures ANOVA, Dunnett’s two-sided multiple-comparison test with control was used for statistical evaluation of data with normal distribution. Kruskal–Wallis Z test (Dunn’s test) was used for data not meeting the assumptions of normality. Asterisks indicate statistically significant differences ***P≤0.001; **P≤0.01; *P≤0.5; (*)*P*≤0.1; ns, not significant.

To understand the contrasting behaviour of our mutants under *in vitro* and *ex vitro* conditions, as well as in comparison with previously reported results for ssp. *andigena*, we examined whether it could be linked to different gene expression patterns. Interestingly, leaf *BEL11* transcripts levels were significantly lower in all four RNAi lines under both conditions (Figs 1A, 4A).

Although leaf *BEL11* transcript levels of *ex vitro* plants were reduced in all RNAi lines and this decrease was apparent in phloem sap directed to below-ground organs, particularly in lines with the most suppressed tuberization *ex vitro* (Fig. 4J), *BEL11* transcript levels in stolons exhibited no significant differences (Fig. 4D). However, the levels in roots indicated a decreasing trend, particularly in line 10 (Fig. 4G).

Due to the reported ability of BEL11 to compete with BEL5 and BEL29 for the dimerization partner, POTH1, we further determined their transcripts levels to estimate their potential mutual inter-relationships. Unexpectedly, leaf *BEL5* transcript levels significantly decreased in three RNAi lines (line 1, 10, and 12), and line 19 showed a decreasing trend (Fig. 4B). Intriguingly, the decrease in *BEL11* appeared to be stronger, leading to a slight (insignificant) imbalance in the *BEL5/BEL11* transcript ratio in favour of *BEL5* (Supplementary Fig. S5E). In stolons and roots, no significant changes in *BEL5* transcript levels were observed (Fig. 4E, H). Similarly, *POTH1* and *BEL29* transcript levels did not differ significantly from those of the WT in any of the tested organs (Supplementary Fig. S5A–D). Furthermore, we evaluated transcript levels of the tuberization inducer SP6A and, surprisingly, did not find the expected correlation with the phenotype of suppressed tuberization; no significant decrease in *SP6A* levels was observed in any organ, but rather a tendency towards increased levels, particularly in lines 10 and 12 (Fig. 4C, F, I).

### BEL11 down-regulation improved photosynthetic performance and changed biomass allocation

**Fig. 5.**
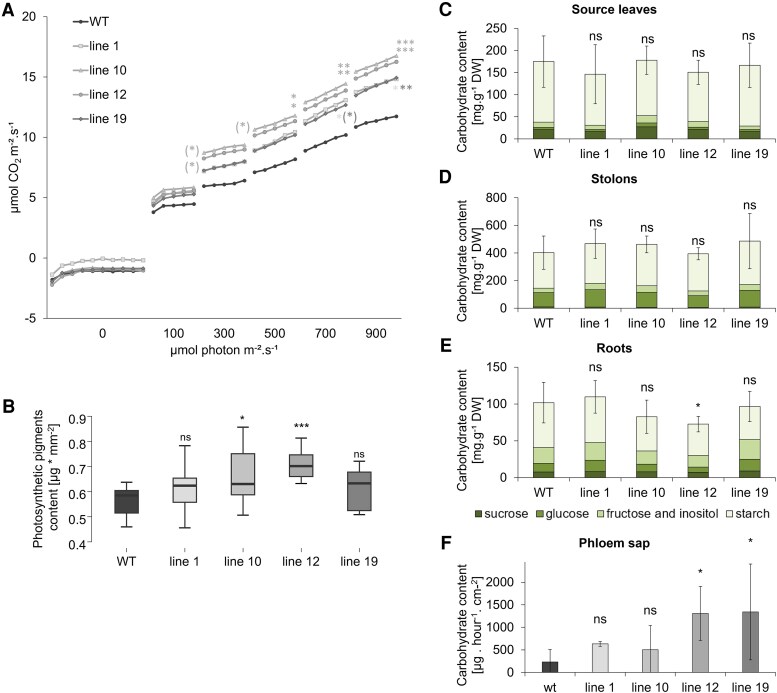
Photosynthetic parameters and carbohydrate contents. (A) Net photosynthesis rate under different irradiances. (B) Photosynthetic pigment contents (sum of chl *a*, chl *b*, and carotenoids). Carbohydrate contents in (C) source leaves, (D) stolons, (E) roots, and (F) phloem sap in *ex vitro* plants cultivated in soil, (A–E) in the cultivation chamber, or (F) in the greenhouse, under an LD photoperiod and evaluated 21 DAT. WT and four independent BEL11 RNAi lines: 1, 10, 12, and 19; *n*=3–18; for (C–F), error bars represent the standard deviations calculated for total carbohydrate contents. One-way ANOVA, Dunnett’s two-sided multiple-comparison test with control was used for statistical evaluation of data with normal distribution; Kruskal–Wallis Z test (Dunn’s test) was used for data not meeting the assumptions of normality. Asterisks indicate statistically significant differences ***P≤0.001; **P≤0.01; *P≤0.05; (*)*P*≤0.1; ns, not significant; in the case of (A) for the last measurement at a given irradiance. For statistical analysis of individual saccharides in the spectra, see Supplementary Table S3.

As nodal segments cultured in darkness depend entirely on an external carbohydrate supply from a high-sucrose medium, while *ex vitro*/tuber-derived plants rely on their own assimilate production, we further investigated whether the contradictory tuberization results could be ascribed to different ways to gain and distribute carbon and energy. In soil-cultivated plants, we found substantial differences in several photosynthetic characteristics. Source leaves of all four RNAi lines exhibited significantly higher net photosynthetic rates compared with the WT already under lower irradiance (300 µmol photons m^−2^ s^−1^) and, with increasing irradiance, the differences were even more pronounced (Fig. 5A). Notably, lines 10 and 12 with the strongest tuberization phenotype (weakest tuberization *ex vitro*) displayed the most prominent increases in net photosynthetic rates and exhibited higher contents of photosynthetic pigments (Fig. 5B; Supplementary Fig. S6) with non-impaired maximum quantum yield of chl *a* fluorescence (Supplementary Fig. S6).

To further understand the reason for a non-impaired and even an enhanced photosynthetic rate in BEL11 RNAi lines, we determined the carbohydrate contents in tissues and phloem exudates and characterized allocation of carbohydrates to source and sink organs. Surprisingly, in all of the tested organs (leaves, stolons, and roots), the carbohydrate content per unit of dry matter did not systematically differ among genotypes, either in soil or in hydroponics (Fig. 5C–E; Supplementary Fig. S7). Although there was high variability in measured values of carbohydrates in phloem sap that showed statistical significance only in two lines (Fig. 5F), we repeatedly recorded a clear tendency towards increased levels in all RNAi lines compared with the WT (Supplementary Fig. S8). The carbohydrate composition of the phloem sap, comprising predominantly sucrose, showed no significant differences among genotypes (77–89%).

The higher photosynthetic capacity but unchanged carbohydrate content in tissues, along with a slight increase in carbohydrate contents in phloem exudates in RNAi lines, led us to hypothesize that the difference may lie in biomass allocation. We analysed non-tuberizing plants (LD, 21 DAT) cultivated *ex vitro* in hydroponics (enabling us to harvest the complete root biomass). The total biomass and leaf area of all four RNAi lines were comparable with those of the WT (Supplementary Fig. S9), but lines 10 and 12 with the strongest tuberization phenotype showed higher ‘underground’ biomass allocation (‘underground’ portion of the shoot, stolons, and roots). This difference resulted from a significantly higher proportion of root biomass in lines 10 and 12, and a trend towards increased root biomass observed in the other two lines (Supplementary Fig. S9). Moreover, significantly enhanced biomass allocation to roots in lines 10 and 12 was also observed in photoautotrophically cultivated *in vitro* plants, reaching 26% and 31% of total biomass, respectively, compared with 21% in the WT. In contrast to roots, all four transgenic lines showed a significantly decreased allocation to stolons compared with the WT (Supplementary Fig. S9). Accordingly, the relative underground biomass allocation to roots over stolons was significantly higher in all RNAi lines (79–96%, compared with 64% in the WT; Supplementary Fig. S9).

### BEL11 RNAi lines with the strongest phenotype kept distinct growth dynamics, and carbohydrate and transcript patterns even at the stage of tuberization onset

**Fig. 6.**
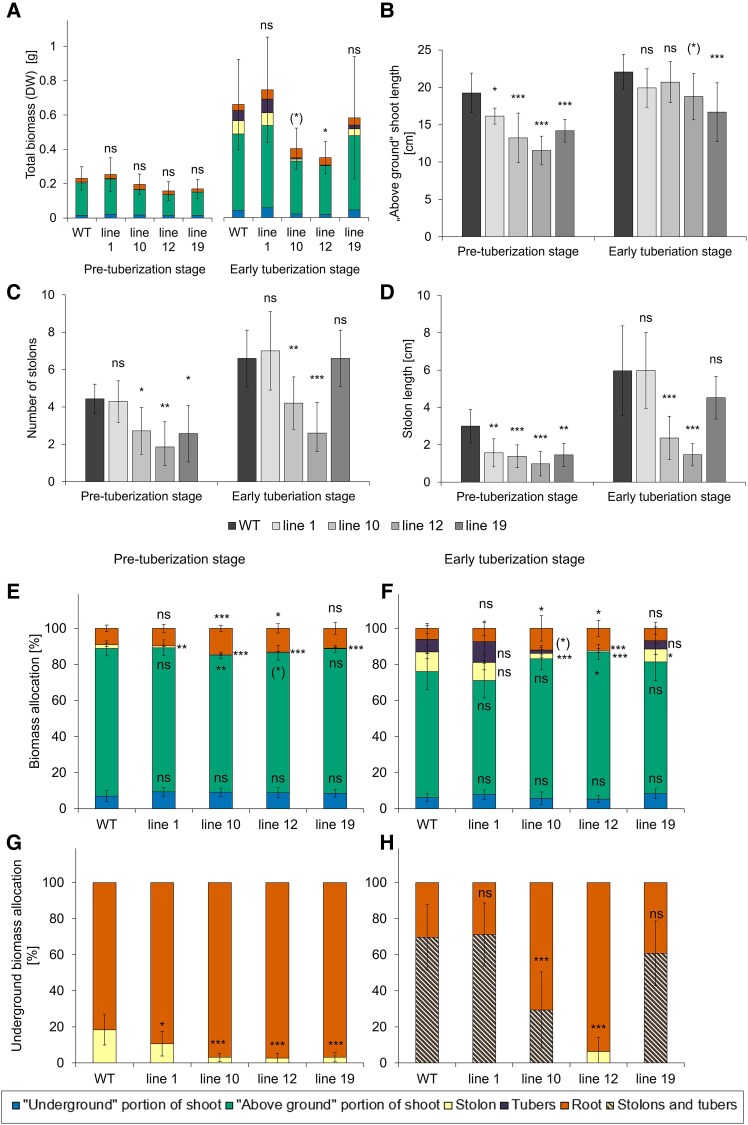
Growth characteristics at the pre-tuberization and early tuberization stages. (A) Total DW biomass, (B) average ‘above-ground’ shoot length, (C) average number of stolons, (D) average stolon length, (E and F) pattern of DW biomass allocation in pre- and early tuberization stage, respectively, and (G and H) pattern of DW biomass allocation in ‘underground’ sinks in the pre- and early tuberization stage, respectively. Plants were cultivated in a hydroponic system under an SD photoperiod and evaluated at the pre-tuberization stage (28 DAT) and early tuberization stage (41 DAT). WT and four independent BEL11 RNAi lines: 1, 10, 12, and 19; *n*=7–11. Error bars represent the standard deviation (A; calculated for total biomass). One-way ANOVA, Dunnett’s two-sided multiple-comparison test with control was used for statistical evaluation of data with normal distribution; Kruskal–Wallis Z test (Dunn’s test) was used for data not meeting the assumptions of normality. Asterisks indicate statistically significant differences ***P≤0.001; **P≤0.01; *P≤0.05; (*)*P*≤0.1; ns, not significant.

To better understand the role of BEL11 in the link between tuberization and metabolic and growth responses, we further followed the course of change during two developmental stages under tuber-promoting SDs: the pre-tuberization stage (28 DAT) and the early tuberization stage (41 DAT). Similarly to other *ex vitro* experiments, tuber yields were significantly reduced in mutant lines 10 and 12, and line 19 showed a similar trend (Supplementary Fig. S10). At the pre-tuberization stage, most of the growth response shifts in transgenic lines resembled those observed in non-tuberizing plants under LDs (Fig. 6; for comparison, see Supplementary Fig. S9). At the early tuberization stage, the results were slightly different. All transgenic lines produced more or less similar shoots with a comparable number of nodes (Fig. 6B; Supplementary Fig. S11B, similar to non-tuberizing plants), in lines 10 and 12 accompanied by a smaller leaf area (Supplementary Fig. S11A) and reduced total biomass (Fig. 6A). Importantly, lines 10 and 12 maintained the underground phenotype distinct from that of the WT as observed in other experiments. In these lines, we again observed a higher proportion of roots (Fig. 6F) and a lower proportion of stolon biomass (both decreased number and length) (Fig. 6C, D, F). Accordingly, they also reduced biomass allocation to tubers (Fig. 6F) and exhibited a significant allocation shift favouring roots over stolons and tubers (Fig. 6H).

**Fig. 7.**
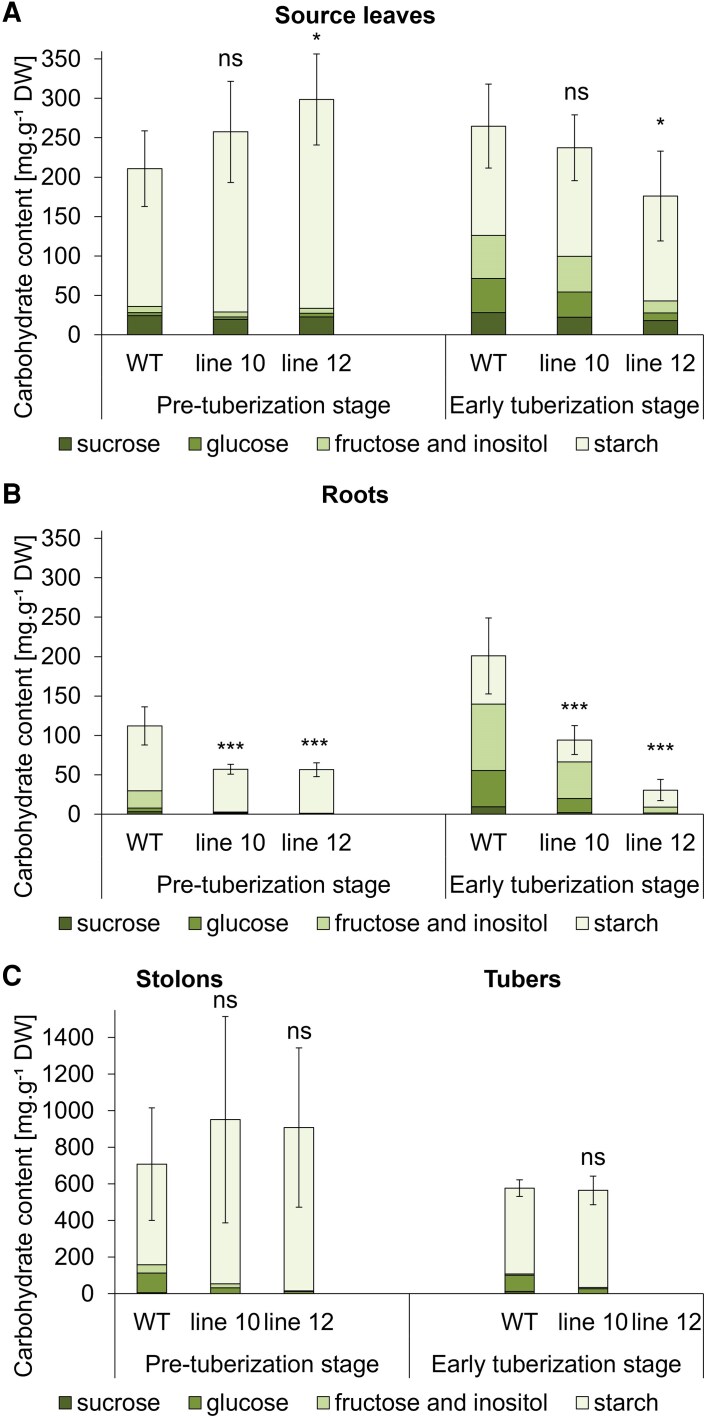
Carbohydrate contents in individual organs at the pre-tuberization (28 DAT) and early tuberization stages (41 DAT) in a hydroponic system under a SD photoperiod. WT and two independent BEL11 RNAi lines: 10 and 12; *n*=3–6. Error bars represent the standard deviations calculated for total carbohydrate contents. One-way ANOVA, Dunnett’s two-sided multiple-comparison test with control was used for statistical evaluation. Asterisks indicate statistically significant differences ***P≤0.001; *P≤0.05; ns, not significant. For statistical analysis of individual saccharides in the spectra, see Supplementary Table S5.

**Fig. 8.**
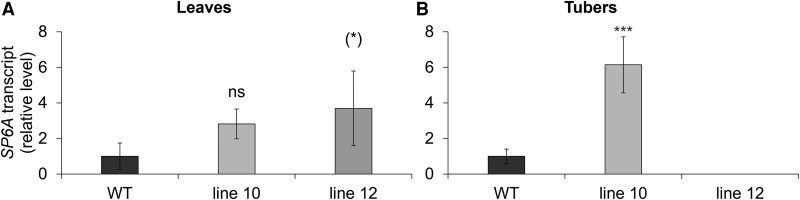
*SP6A* transcript levels at the early tuberization stage. (A) Leaves and (B) tubers. RNA was isolated from plants cultivated in a hydroponic system under an SD photoperiod and evaluated at the early tuberization stage (41 DAT). WT and two independent BEL11 RNAi lines: 10 and 12; normalized to the copy number of the reference transcript—*polyubiquitin*. Three technical replicates were performed per cDNA dilution (5×). The WT level was set to 1 (mean Δ Ct values for the WT are provided in Supplementary Table S2). *n*=3–4. Error bars represent the standard deviations. One-way ANOVA, Dunnett’s two-sided multiple-comparison test with control was used for statistical evaluation. Asterisks indicate statistically significant differences ***P≤0.001; (*)*P*≤0.1; ns, not significant.

Given the more pronounced phenotype during the transition to tuber onset observed in lines 10 and 12, we selected them for further analyses. Notably, we observed significantly lower carbohydrate content per unit of DW in roots at both pre-tuberization and early tuberization stages; particularly noticeable were lower starch levels in transgenics (Fig. 7B; Supplementary Table S5). This demonstrates a clear enhancement of this trait, compared with non-tuberizing individuals (Fig. 5E). Importantly, assimilate transport towards underground organs does not seem to be compromised at any developmental stage; we even found a trend of elevated carbohydrate levels in the phloem sap (Supplementary Fig. S12). These findings indicate that carbohydrates are utilized for growth instead of being stored in roots, resulting in promoted root growth. In leaves, total carbohydrate levels remained unchanged (Fig. 7A). The total levels in stolons and tubers varied markedly across individual lines, with these differences not being significantly different among genotypes (Fig. 7C). Nevertheless, an upward trend in starch level was found in transgenic stolons at the pre-tuberization stage (Supplementary Table S5). Leaf *BEL11* transcript levels predictably decreased in transgenic lines (Supplementary Fig. S13), while, consistent with our previous findings, *SP6A* transcript levels did not correlate with the suppressed tuberization in transgenics; in fact, they exhibited an upward trend in leaves and even a significant increase in young tubers of line10 (Fig. 8).

Altogether, biomass allocation in the BEL11 RNAi lines differed significantly from that in the WT, with reduced elongation of above-ground stems, suppressed stolon and tuber formation, and enhanced root growth associated with lower carbohydrate content per unit of DW. These alterations became particularly pronounced with the onset of tuberization in lines 10 and 12, which exhibited the most suppressed tuberization, accompanied by an unexpected tendency towards increased *SP6A* transcript levels.

## Discussion

In our study, we analysed the effects of down-regulation of the transcription factor BEL11 on tuberization in cultivated potato ssp. *tuberosum*. On nodal segments *in vitro*, all tested parameters of tuber induction were promoted in all BEL11 RNAi lines (Fig. 1), thus supporting the negative regulatory role of BEL11 in tuberization, as reported earlier for ssp. *andigena* (Ghate *et al*., 2017). However, under *ex vitro* conditions, we revealed a marked contrast as none of the BEL11 RNAi lines showed enhanced tuberization. In fact, the lines with the strongest *in vitro* tuberization (lines 10 and 12) exhibited delayed tuberization and significantly reduced total tuber weights *ex vitro* compared with the WT (Fig. 2). In tuber-derived plants, the tuberization suppression was even stronger, showing reduced tuber numbers and dramatically lower yields across all RNAi lines (Fig. 3). This substantially contradicts the results of Ghate *et al*. (2017), who reported considerably higher tuber yields in *ex vitro* soil-grown BEL11 RNAi lines of ssp. *andigena*. Surprisingly, until now, only a few studies have compared tuberization phenotypes under *in vitro* and *ex vitro* conditions. For example, Rosin *et al*. (2003) and Kondhare *et al*. (2021) found that ssp. *andigena*, requiring SD conditions for tuberization in soil, was able to tuberize under LDs when supplied with sufficient exogenous sucrose *in vitro*. Differences between tuberization under natural and *in vitro* conditions probably reflect a distinct source–sink balance under both these conditions. Intact *ex vitro*/tuber-derived plants, which rely on their own assimilate production, exhibit a markedly more complex source–sink interaction. The discrepancy in the role of BEL11 in the regulation of tuberization between subspecies *andigena* and *tuberosum* can be attributed to their distinct evolutionary histories. Potatoes originated in the Andean region and later expanded globally, requiring adaptations to various daylengths and temperatures (Hardigan *et al*., 2017; Gutaker *et al*., 2019). Unlike the strictly SD ssp. *andigena*, modern ssp. *tuberosum* cultivars can tuberize under LDs, although tuberization is accelerated under SDs. One example of a molecular adaptation of modern cultivars is the presence of truncated *StCDF1* alleles, which have lost daylength-dependent regulation (Kloosterman *et al*., 2013). Moreover, domestication led to substantial changes in the source–sink relationships associated with a dramatic strengthening of the allocation of assimilates to developing tubers and, in addition, there are orders of magnitude differences in yields between them (e.g. Spooner and Hetterscheid, 2005). Altogether, the data indicate that the impact of BEL11 on tuberization strongly depends on the particular potato genotype (subspecies) and specific environmental context.

To clarify the observed phenomena, we focused on the analysis of expression patterns (Fig. 4). *BEL11* transcript levels were significantly reduced in leaves of all RNAi lines regardless of cultivation conditions. Although a tendency for lower levels of *BEL11* transcript was also detected in the phloem sap and roots (both particularly prominent in lines with the most distinct tuberization phenotypes), surprisingly, no such reduction was mirrored in stolons and tubers grown *ex vitro*, where *BEL11* levels remained unchanged. The discrepancy between individual organs may be caused by different efficiencies of *BEL11* silencing, for example due to variations in CaMV *35S* promoter activity driving *BEL11AS* expression (Rahamkulov and Bakhsh, 2020) or decreased *BEL11* transcript accessibility to degradation by the RNAi machinery, probably resulting from the interaction with RNA-binding proteins (Cho *et al*., 2015). Since these factors can influence the effect of BEL11 in an organ-/tissue-specific manner, the interpretation of the observed phenotypes is rather complicated. In this context, it is also necessary to mention that Ghate *et al*. (2017) observed promoted tuberization in all BEL11 RNAi *andigena* lines without a clear correlation with *BEL11* changes in transcript level in stolons or leaves.

Another issue that needs to be addressed is the interaction between individual BEL transcription factors. Importantly, reduced *BEL11* transcript levels in leaves of the RNAi lines were accompanied by down-regulation of *BEL5*, which is considered a positive regulator of tuberization. This decrease in *BEL5*, which may be connected to the observed phenotype, is undoubtedly not a direct effect of RNAi. The *BEL11* antisense construct covers a unique 401 bp fragment (of the 5'-UTR and exon 1) that has practically no sequence similarity detectable by the Basic Local Alignment Search Tool. BEL transcription factors recognize tandem TTGAC motifs in promoter sequences of their target genes, and the *BEL5* promoter itself contains these motifs (Chen *et al*., 2004; Sharma *et al*., 2014). Therefore, BEL11 might regulate *BEL5* transcription, although the function of BEL11 as a transcription regulator has not been tested yet. Consistently, the transcript pattern of *BEL11* corresponded to that of *BEL5* in other tested organs (roots and stolons), where both showed no significant changes. Additional support comes from Ghate *et al*. (2017), who described increased stolon *BEL5* transcript levels in transgenic *andigena* with strengthened *BEL11* mRNA transport towards stolons. However, understanding the interactions of BELs is further complicated due to a proposed BEL5-positive regulation of its own transcription in stolons through an autoregulatory loop (Lin *et al*., 2013). In addition, the transcript levels of both negative tuberization regulators, *BEL11* and *BEL29*, were up-regulated in the stolons of *andigena* transgenic lines with induced *BEL5* transcription (Sharma *et al*., 2016). Altogether, the data repeatedly indicate that the expression of various *BEL* genes may be mutually connected. This could ensure the fine-tuning of target gene transcription in a tissue- and developmental stage-specific manner, which is essential for optimal plant performance under various environmental conditions (e.g. Niu and Fu, 2022). Competition for the common BEL interaction partner, POTH1, represents another level of hypothetical interactions. Thus, BEL11 might inhibit tuberization by competing with the positive regulator BEL5, as already proposed for *andigena* by Ghate *et al*. (2017). In our mutants, both *BEL11* and *BEL5* transcript levels decreased in the leaves, while *POTH1* levels remained unchanged (Fig. 4; Supplementary Fig. S5). Therefore, we evaluated if changes in expression of these mobile signals could have an impact on the expression pattern of the target transcript *SP6A*, coding for a key positive regulator of tuberization (Navarro *et al*., 2011). Interestingly, at the stage prior to tuberization, we observed an upward trend in *SP6A* transcript levels in leaves, stolons, and roots of the two lines showing the strongest suppression of tuberization (Fig. 4). Surprisingly, this pattern became even more pronounced with the onset of tuberization as, in addition to the persistent increasing trend in leaves, a significant 6-fold increase of *SP6A* transcript level was detected in young transgenic tubers (Fig. 8). These findings, showing that suppressed tuberization in our mutants is not necessarily accompanied by a decrease in *SP6A* levels, suggest a potentially weakened superiority of the SP6A signal within the very complex tuberization signalling network in cultivated potato. Interestingly, in another spontaneously tuberizing potato line (of cv. Lada), we have previously shown that a multi-fold enhanced *SP6A* transcript level was accompanied by accelerated tuber onset, but reduced overall tuber yield (Fischer *et al*., 2008; Ševčíková *et al*., 2017). Taken together, our data suggest that the impact of individual tuberization regulators varies in complex regulatory networks of ssp. *tuberosum* and ssp. *andigena*, which precludes simple predictions of the effects of manipulating individual regulatory components as well as an easy interpretation of observed phenotypes.

Furthermore, given the wide range of downstream targets of BEL5, or indeed of BEL11, they are likely to influence not only tuberization but also various growth and metabolic processes reflected ultimately in tuber yield. Although, in potato, BEL and FT-like signalling beyond tuberization has not been extensively studied, in other species they have been shown to regulate various processes (reviewed by Jin *et al*., 2021; Niu and Fu, 2022). For example, in tomato, a close potato relative, the BEL11 and BEL5 homologues (SlBEL11 and SlBEL2) have been shown to act as regulators of photosynthesis by modulating expression of genes involved in chloroplast development (Meng *et al*., 2018; He *et al*., 2022; Niu *et al*., 2022). Photosynthesis impairment would be a possible explanation for the reduced tuber yields observed under natural-like conditions, while tuberization on nodal segments cultivated *in vitro* on a high-carbohydrate medium was promoted. Interestingly, we observed no drop in carbon assimilation efficiency. Instead, photosynthetic rates and pigment levels were higher in BEL11 RNAi mutants (Fig. 5), suggesting a negative effect of BEL11 (and/or BEL5, which was also down-regulated) on photosynthetic activity, similar to their tomato homologues. Though increased photosynthesis is often associated with enhanced tuber yields (Fernie *et al*., 2020; Rosado-Souza *et al*., 2023), this correlation may not always hold. For instance, potato transgenic lines expressing Arabidopsi*s* Hexokinase 1 in guard cells and *SP6A* under a leaf/stem-specific promoter exhibited reduced photosynthetic assimilation, but achieved higher overall tuber yields (Lehretz *et al*., 2021a).

Beyond the capacity for carbon assimilation itself, downstream processes related to assimilate distribution are equally important, as they ultimately determine biomass allocation to plant organs and therefore affect overall tuber yield (Sonnewald and Fernie, 2018). While we observed no difference in total biomass at the non-tuberizing stage (Supplementary Fig. S9), after the onset of tuberization, the two lines with the most pronounced phenotype showed a reduction, accompanied by smaller leaf area (Fig. 6; Supplementary Fig. S11). This can influence the overall amount of fixed carbon, even if the assimilation efficiency per unit of leaf area was increased. Nevertheless, carbohydrate phloem transport to the underground organs did not appear to be compromised, as indicated by a trend towards increased carbohydrate content in the phloem sap of transgenics at the non-tuberizing as well as the early tuberization stage (Fig. 5; Supplementary Fig. S12), resulting in preferential allocation of assimilates to growth of underground parts in particular (Fig. 6; Supplementary Fig. S9). Typically, in potato, such a pattern of carbon allocation results in simultaneous promotion of root growth and tuberization, for example shown in *SP6A-*overexpressing lines (Lehretz *et al*., 2021b), or in plants grown under low nitrate (e.g. Koch *et al*., 2024). The answer to why our BEL11 RNAi plants did not outperform the WT in tuber yield could lie in their altered biomass allocation among underground organs. In all non-tuberizing RNAi lines, the biomass was preferentially allocated to roots over stolons (Supplementary Fig. S9), and a similar pattern was preserved up to the pre-tuberization stage (Fig. 6). At early tuberization, this pattern further persisted in lines with strongly suppressed tuber formation, accompanied by even lower root carbohydrate levels (Figs 6, 7), indicating prolonged root growth promotion in these transgenics.

Based on the literature data, the cessation of stolon growth occurs concomitantly with the onset of tuberization, but this in itself is not a signal promoting tuber formation (e.g. Struik *et al*., 1988; Xu *et al*., 1998a, b). One possible cause of limited carbon allocation to stolons and tubers might be linked to phloem unloading. In roots, saccharides pass through plasmodesmata and symplasmic unloading is directly connected with root growth (e.g. Liu *et al*., 2024). This would be consistent with our observation of lower root carbohydrate content while biomass accumulation was enhanced, suggesting that utilization for growth may further enhance sink strength. In stolons, unloading is more complicated, starting with the prevailing apoplasmic pathway, where sucrose crosses the membranes by the concerted action of SWEET and SUT transporters, or hexoses (sucrose cleavage products) via hexose transporters (reviewed, for example, by Braun, 2022). During the early stages of tuberization, the unloading shifts to symplasmic mode and later returns to a predominant apoplasmic one during subsequent tuber enlargement (Viola *et al*., 2001). Intervention in *BEL* signalling (modifying transcript levels and mobility together with assimilate flow) can induce changes in gene expression in the underground organs, and consequently alter phloem flow towards roots, underground axillary buds, and stolons. The proposed concept of altered carbohydrate flux towards the sink representing a significant component of the tuberization regulation network was further supported by experiments in a simplified *in vitro* system, where BEL11 RNAi nodal segments with high carbohydrate supply from the medium lacked the competing root sink and exhibited enhanced tuberization. Taken together, the strengthened root growth at the expense of stolons (and subsequently tubers) in BEL11 RNAi lines is likely to be caused by altered sink strength, possibly connected with phloem unloading efficiencies in root and/or stolon tissues. However, understanding the precise molecular mechanism requires further investigation.

If we extend our speculation further and consider an increase in photosynthetic capacity as a primary change stemming from the down-regulation of BEL11, then we can propose the following sequence of events in ssp. *tuberosum* BEL11 RNAi lines. An increase in photosynthetic capacity may generate a signal regarding C/N imbalance and, thus, to strengthen the need for nitrogen uptake, the export of carbohydrates to below-ground organs is reinforced. Enhanced transport of assimilates downwards usually promotes tuberization. However, due to the above-mentioned different unloading mechanisms in stolons and roots, the signal distribution between them differs correspondingly and, combined with considered stolon-specific changes in *BEL* transcription and activity, causes tuberization restriction.

## Conclusion

This study clearly demonstrates substantial differences between the tuberization regulatory network of the cultivated potato variety (ssp. *tuberosum*) and the experimental potato model of the *andigena* group, emphasizing the impossibility of directly transferring knowledge from one system to another. During domestication selecting for maximal tuber yields, weakening the strength of certain regulatory factors (such as photoperiod) probably increased the strengths of other signals (generated by metabolic balance and providing indispensable information on the plant energetic status) within the complex network governing tuberization. Thus, factors that exhibit a negative function in ssp. *andigena* may have lost this role in the cultivated potato due to the selective pressure. BEL11 could be an example, as it negatively impacts tuber yields in ssp. *andigena*; however, in cultivated potato, this negative regulatory function appears to be suppressed or even reversed.

## Supplementary Material

eraf551_Supplementary_Data

## Data Availability

The raw datasets are freely available in the Zenodo repository: https://doi.org/10.5281/zenodo.17779108 (Zounková *et al*., 2025).

## Supplementary data

The following supplementary data are available at *JXB* online.

Fig. S1. Selection of BEL11 RNAi transgenic lines and tuberization *in vitro.*

Fig. S2. Tuberization *ex vitro* in hydroponics.

Fig. S3. Tuberization *ex vitro* in the cultivation chamber and greenhouse.

Fig. S4. Tubers harvested from *ex vitro* plants cultivated in the greenhouse.

Fig. S5. Selected transcript levels.

Fig. S6. Selected photosynthetic parameters.

Fig. S7. Tissue carbohydrate contents *ex vitro* in hydroponics under LDs.

Fig. S8. Carbohydrate content in phloem exudates *ex vitro* under LDs.

Fig. S9. Selected growth characteristics in non-tuberizing plants.

Fig. S10. Photographs of plants during tuberization onset.

Fig. S11. Shoot characteristics at the pre-tuberization and early tuberization stages.

Fig. S12. Carbohydrate content in phloem exudates during tuberization onset.

Fig. S13. *BEL11* transcript levels at the early tuberization stage.

Table S1. List of primers.

Table S2. Overview of RT–qPCR assay parameters for the WT.

Tables S3–S5. The statistical significance of the differences in the levels of individual carbohydrates

## Abbreviations

DAT: days after transfer
LD: long day
POTH1: POTATO HOMEOBOX 1
SD: short day
SP6A: SELF-PRUNING 6A
WT: wild type
