# Supplementary material for: The role of transcription factor StBEL11 in carbon allocation and tuberization in cultivated potato differs from that known for the model Andean genotype
Source: J Exp Bot. 2025 Dec 24;77(6):1773–88. doi: 10.1093/jxb/eraf551 (PMC13017113; doi:10.1093/jxb/eraf551)
Supplement: eraf551_Supplementary_Data [file eraf551_supplementary_data.zip › jexbot316721-file001.pdf]

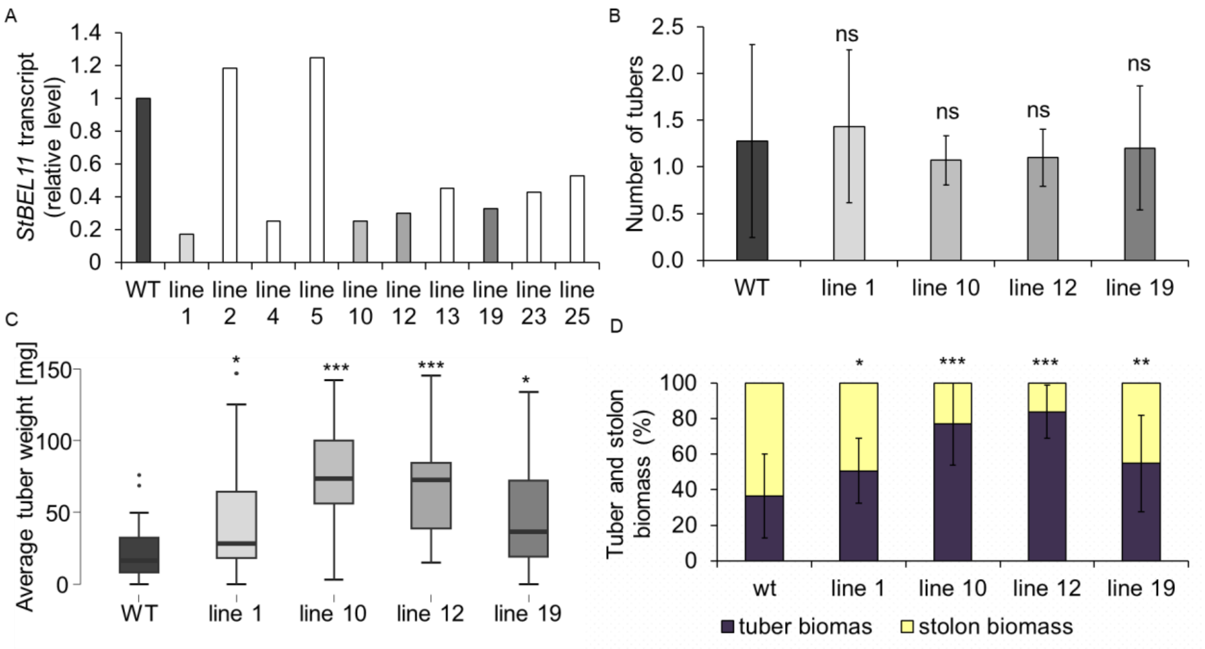

Fig. S1: Selection of BEL11 RNAi transgenic lines and tuberization *in vitro*: (A) screen of leaf *BEL11* transcript levels in ten independent transgenic lines; lines 1, 10, 12 and 19 were selected for further evaluation (Fig. 1A) and subsequent phenotypic analyses; 4-week-old *in vitro* cultivated plants under LD photoperiod; normalized to the copy number of the reference transcript – polyubiquitin; WT was set to 1; 3 technical replicates per each cDNA dilution (5x and 50x). (B) number of tubers; (C) tuber weight; (D) pattern of tuber and stolon biomass allocation; calculated per segment; node cuttings cultivated *in vitro* on TIM under darkness; evaluated 35 DAT; WT and four independent BEL11 RNAi lines: 1, 10, 12 and 19; for (B, C and D) n= 28-30. For B and D error bars represent the standard deviations; ANOVA-One-Way Analysis of Variance, Dunnett's Two-Sided Multiple-Comparison Test With Control was used for statistical evaluation; asterisks indicate statistically significant differences *** (α= 0,001); ** (α = 0.01); * (α= 0,05); ns (not significant).

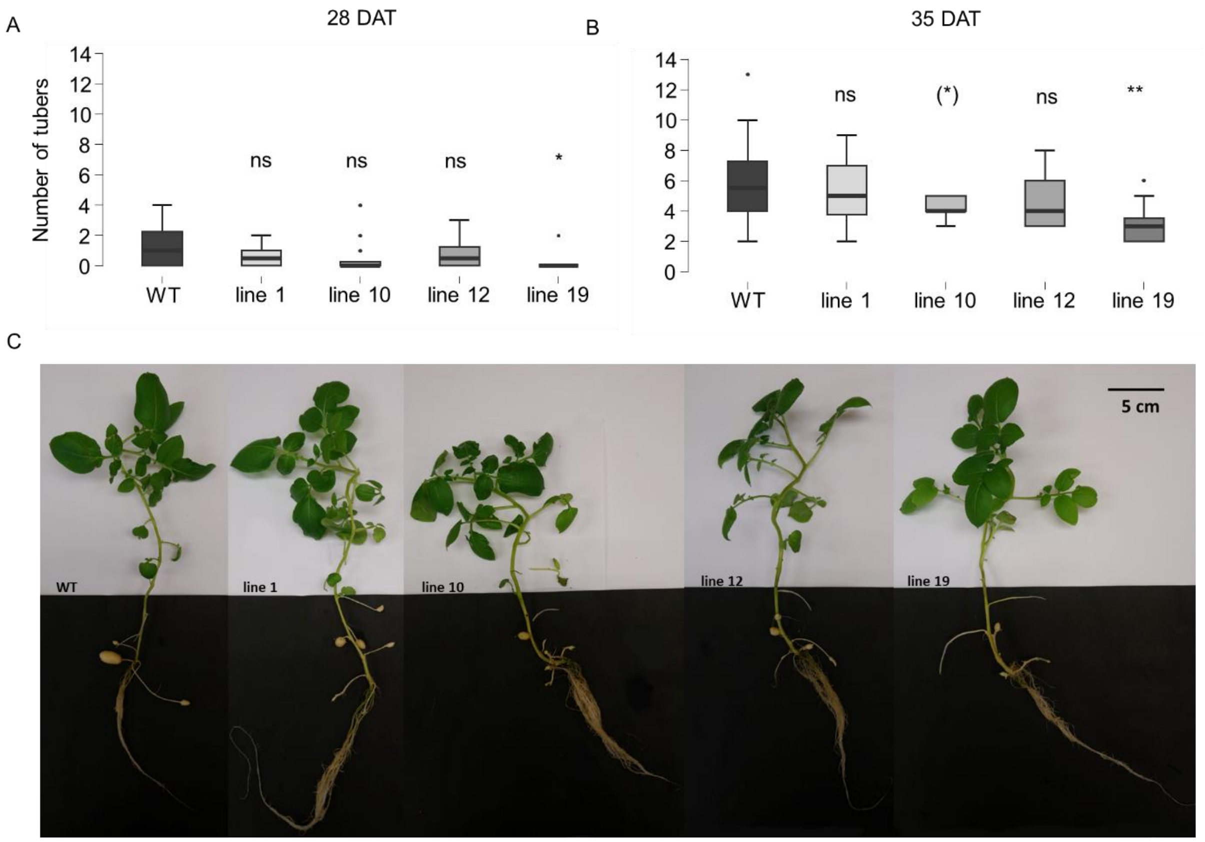

Fig. S2: Tuberization *ex vitro* in hydroponics: average number of tubers per plant (A) 28 DAT and (B) 35 DAT; (C) appearance of tuberizing plants 35 DAT; cultivated in hydroponic system under SD photoperiod; WT and four independent BEL11 RNAi lines: 1, 10, 12 and 19; n= 11-12. ANOVA-One-Way Analysis of Variance, Dunnett's Two-Sided Multiple-Comparison Test With Control was used for statistical evaluation; asterisks indicate statistically significant differences ** (α =0.01); * (α = 0.05); (*) (α = 0.1); ns (not significant).

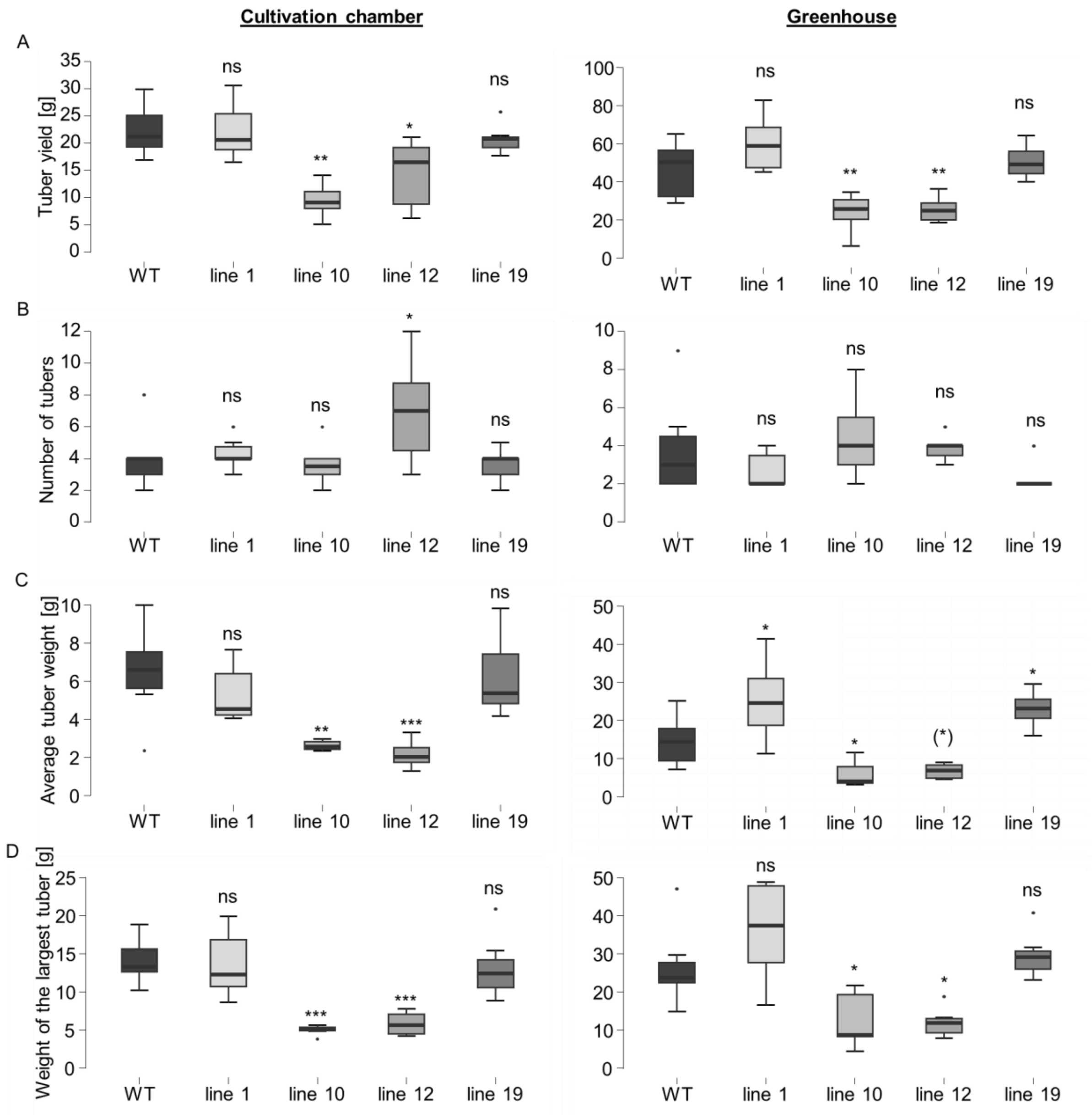

Fig. S3: Tuberization *ex vitro* in the cultivation chamber and greenhouse: (A) average tuber yield; (B) average number of tubers; (C) average tuber weight and (D) average weight of the largest tuber; calculated per plant; soil-cultivated plants under LD photoperiod, evaluated 130 DAT, WT and four independent BEL11 RNAi lines: 1, 10, 12 and 19; n= 6-7. ANOVA-One-Way Analysis of Variance, Dunnett's Two-Sided Multiple-Comparison Test With Control was used for statistical evaluation; asterisks indicate statistically significant differences *** (α = 0.001); ** (α = 0.01); * (α = 0.05); (*) (α = 0.1); ns (not significant).

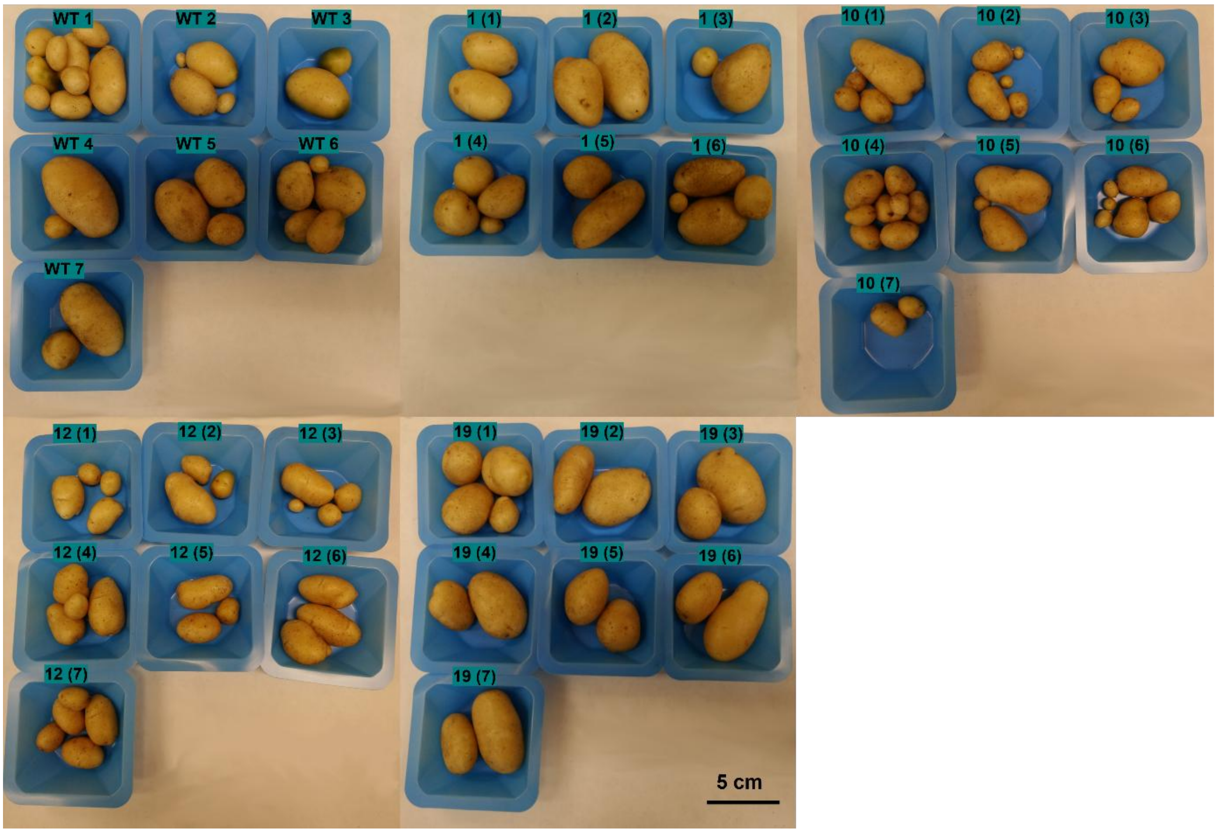

Fig. S4: Tubers harvested from *ex vitro* plants cultivated in the greenhouse: Soil-cultivated plants under LD photoperiod, evaluated 130 DAT, WT and four independent BEL11 RNAi lines: 1, 10, 12 and 19; n= 6-7.

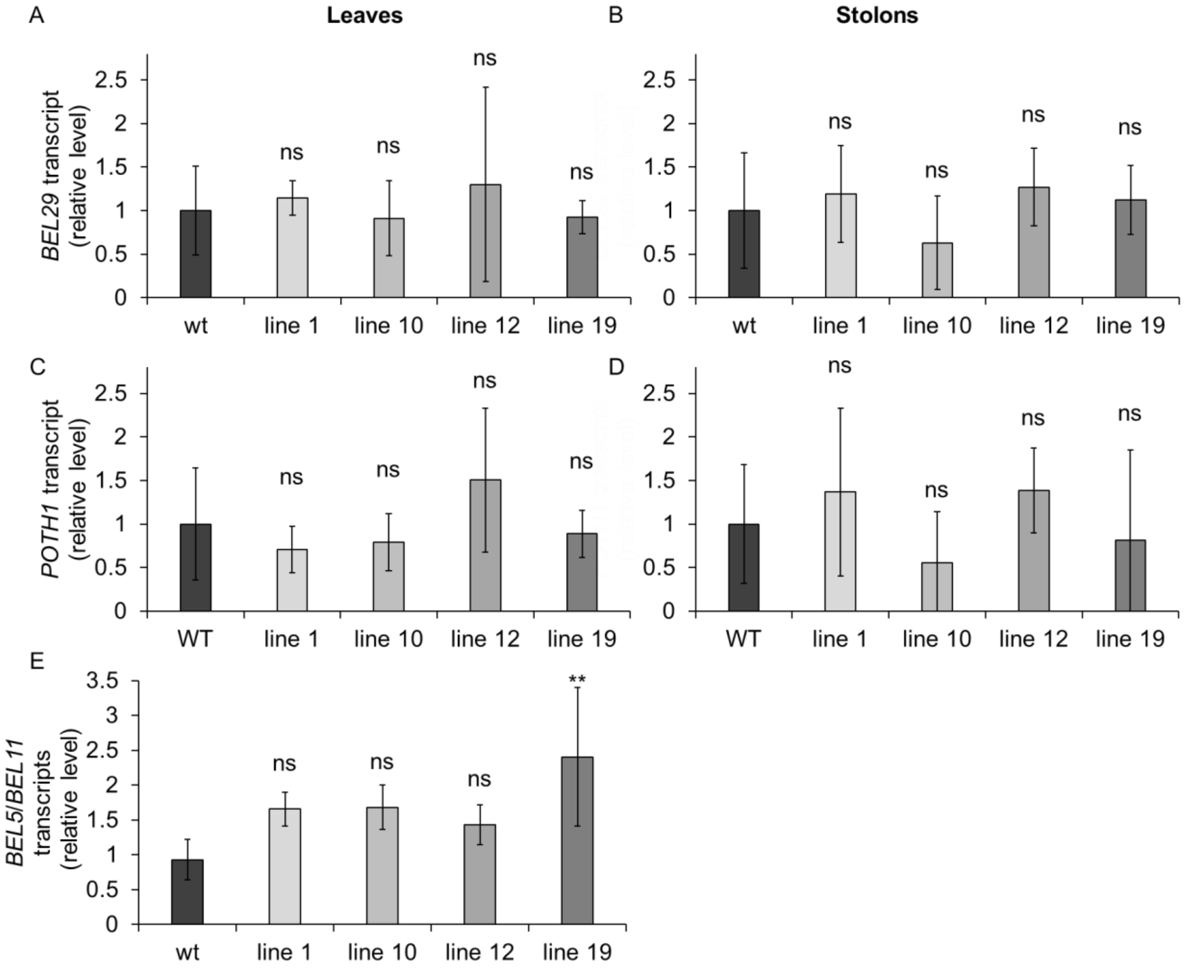

Fig. S5: Selected transcript levels: (A and B) *BEL29*, (C and D) *POTH1,* (E) *BEL5*/*BEL11* transcripts ratio. RNA was isolated from soil *ex vitro* cultivated plants (cultivation chamber) under LD photoperiod; evaluated 21 DAT, WT and four independent transgenic BEL11 RNAi lines: 1, 10, 12 and 19; normalized to the copy number of the reference transcript – polyubiquitin, 3 technical replicates per each cDNA dilution (5x and 50x); for A-E WT level was set to 1 (mean Δ Ct values for WT are provided in Supplementary Table S2). n=3-6; error bars represent the standard deviations; ANOVA-Repeated Measures Analysis of Variance, Dunnett's Two-Sided Multiple-Comparison Test With Control was used for statistical evaluation; asterisks indicate statistically significant differences ** (α = 0.01), ns (not significant).

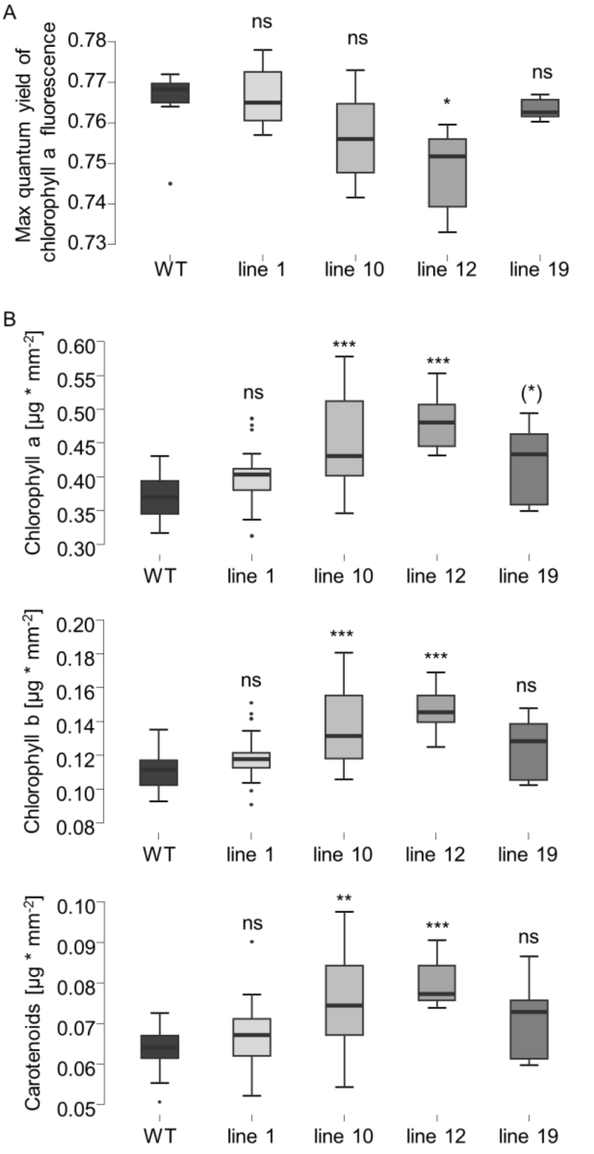

Fig. S6: Selected photosynthetic parameters: (A) maximum quantum yield of chlorophyll a fluorescence [(Fm-Fo)/Fm], (B) photosynthetic pigment contents, measured in soil *ex vitro* cultivated plants (cultivation chamber) under LD photoperiod; evaluated 21 DAT, WT and four independent BEL11 RNAi lines: 1, 10, 12 and 19; n= 5-18. ANOVA-One-Way Analysis of Variance, Dunnett's Two-Sided Multiple-Comparison Test With Control was used for statistical evaluation; asterisks indicate statistically significant differences *** (α= 0.001); ** (α= 0.01); * (α = 0.05); (*) (α= 0.1); ns (not significant); in case of A for the last measurement at given irradiance.

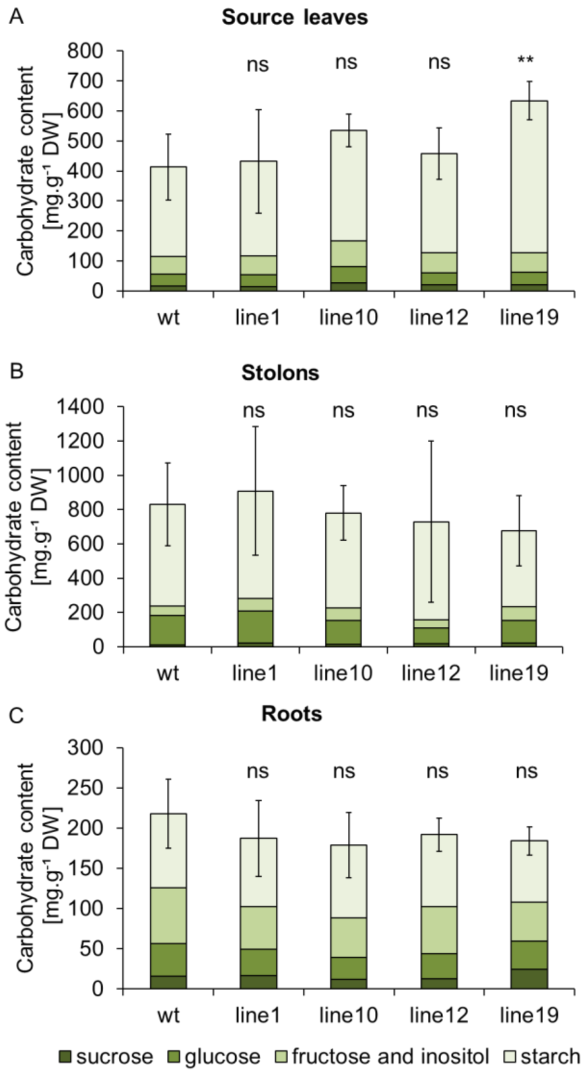

Fig. S7: Tissue carbohydrate contents: in (A) source leaves, (B) stolons and (C) roots, measured in *ex vitro* plants cultivated in hydroponics under LD photoperiod; evaluated 21 DAT, WT and four independent BEL11 RNAi lines: line 1, 10, 12 and 19; n= 6-7; error bars represent the standard deviations for total carbohydrate contents; ANOVA-One-Way Analysis of Variance, Dunnett's Two-Sided Multiple-Comparison Test With Control was used for statistical evaluation; asterisks indicate statistically significant difference ** (α= 0.01); ns (not significant). For statistical analysis of individual saccharides in the spectra see Supplementary Table S4.

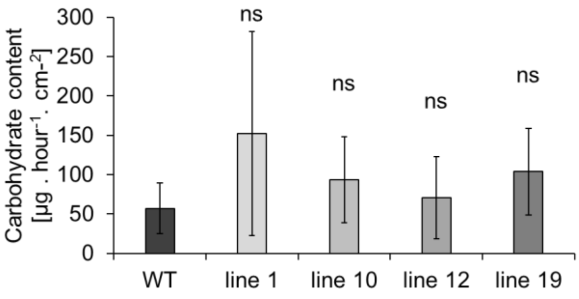

Fig. S8: Carbohydrate content in phloem exudates: Total carbohydrate content related to leaf area; measured in soil cultivated *ex vitro* plants (in the greenhouse) under LD photoperiod; evaluated 21 DAT, WT and four independent BEL11 RNAi lines: line 1, 10, 12 and 19; n= 5-7; error bars represent the standard deviations; Kruskal-Wallis Z test (Dunn's test) was used for statistical evaluation; ns (not significant).

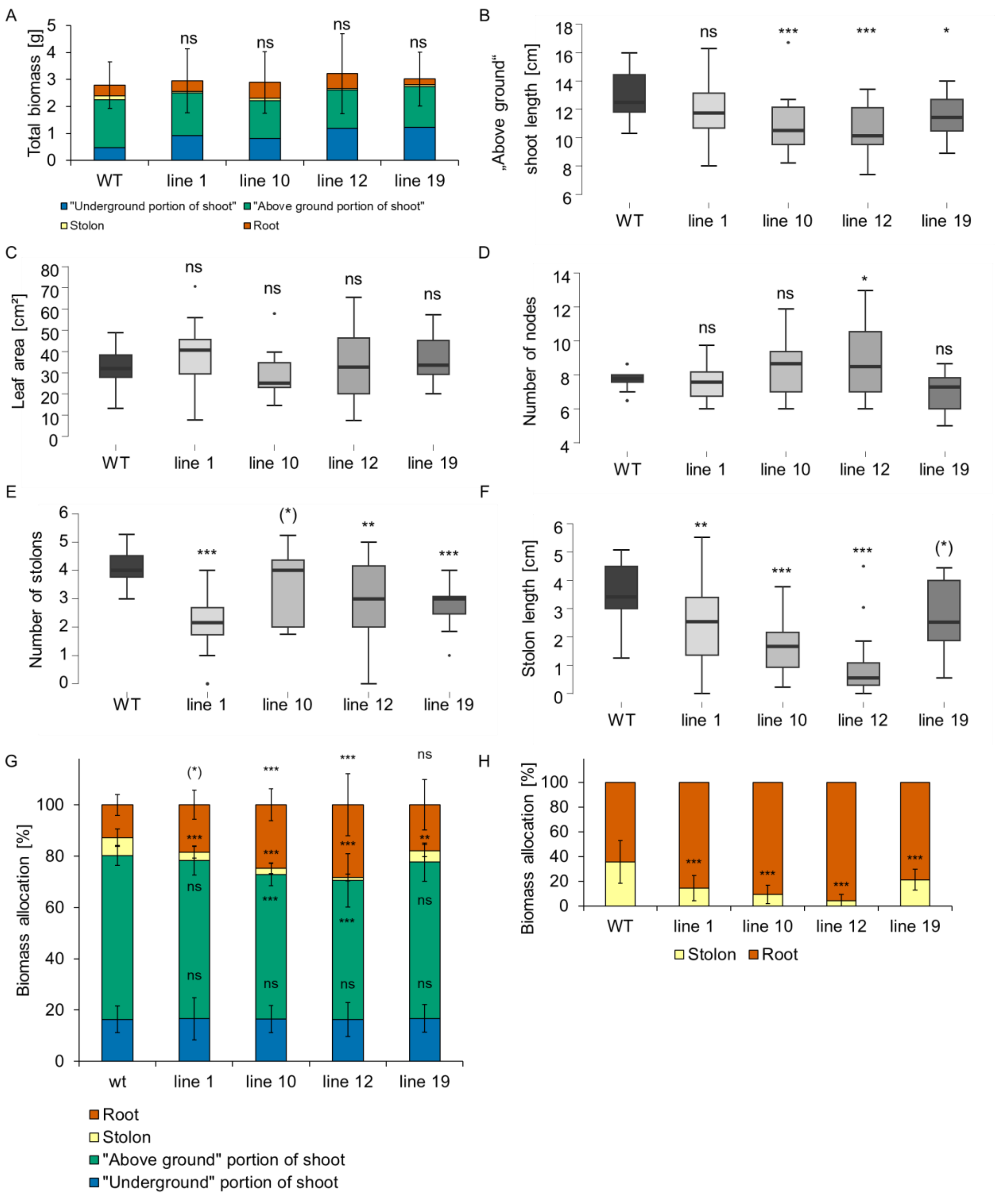

Fig. S9: Selected growth characteristics in non-tuberizing plants: (A) total FW biomass, (B) "above ground" shoot length, (C) leaf area, (D) number of above-ground nodes, (E) number of stolons, (F) stolon length, (G) pattern FW biomass allocation, (H) pattern of FW biomass allocation in "underground" sinks; calculated per plant; plants cultivated in hydroponic system under LD photoperiod; evaluated 21 DAT, WT and four independent BEL11 RNAi lines: 1, 10, 12 and 19; n= 22-24. For A, G and H error bars represent the standard deviations (in case of A calculated for total biomass); ANOVA-One-Way Analysis of Variance, Dunnett's Two-Sided Multiple-Comparison Test With Control was used for statistical evaluation; asterisks indicate statistically significant differences *** (α =0.001); ** (α =0.01); * (α =0.05); (*) (α = 0.1); ns (not significant).

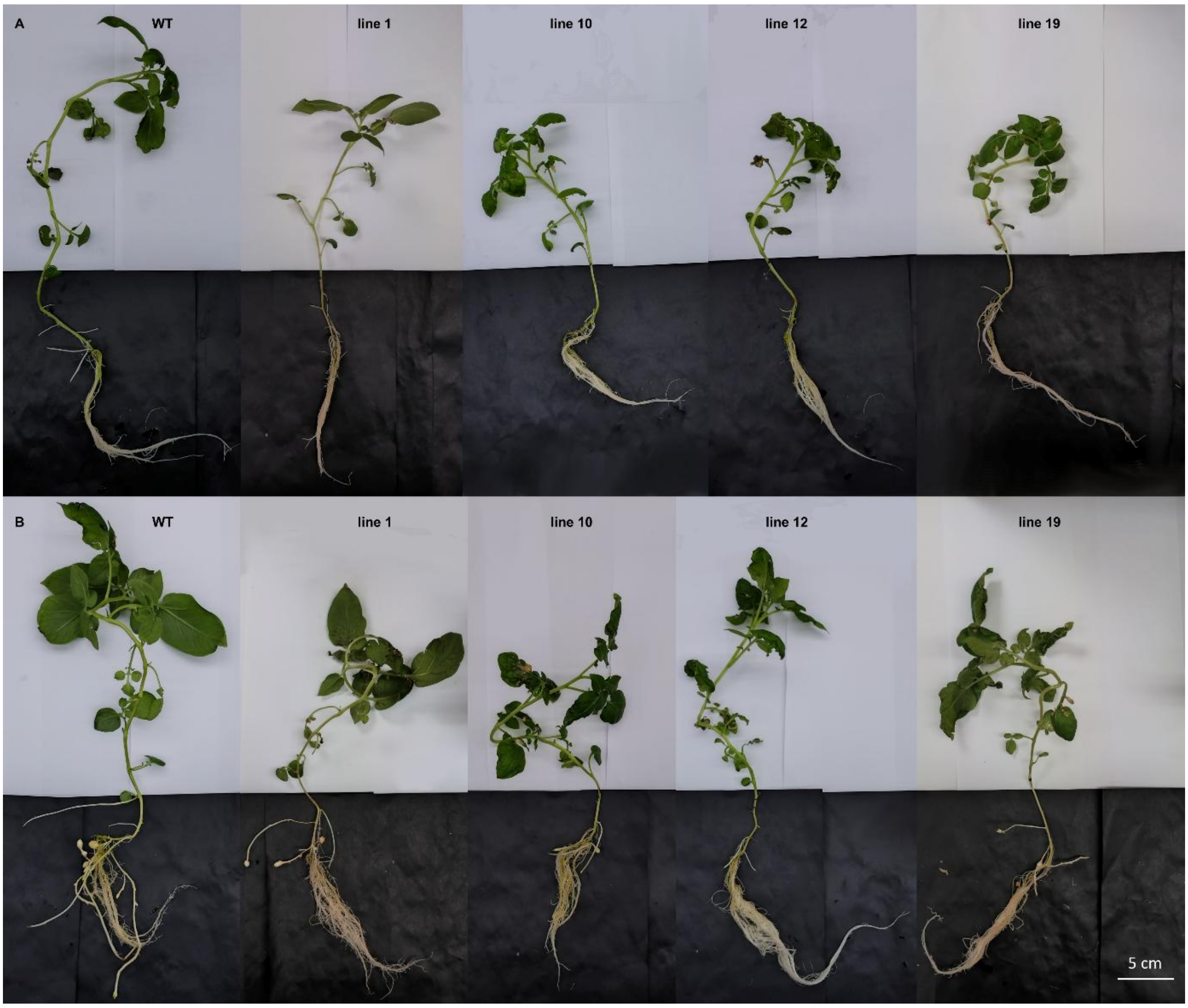

Fig. S10: Photos of plants during tuberization onset: (A) pre-tuberization stage (28 DAT) and (B) early tuberization stage (41 DAT); plants cultivated in hydroponic system under SD photoperiod; WT and four independent BEL11 RNAi lines:1, 10, 12 and 19.

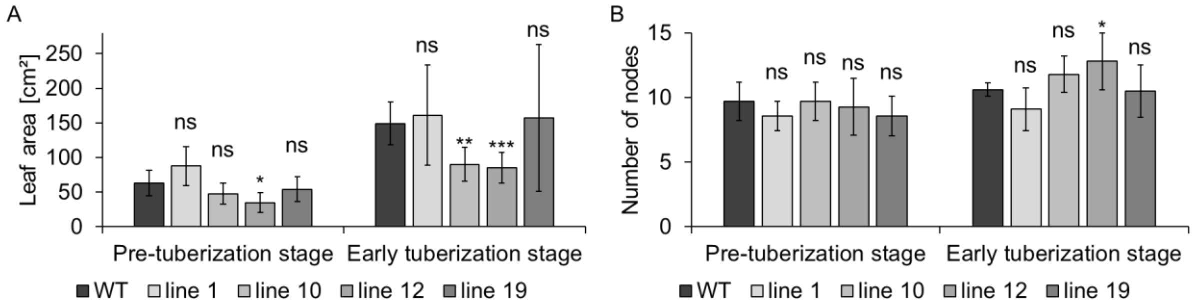

Fig. S11: Shoot characteristics at the pre-tuberization and early tuberization stages: (A) leaf area, (B) number of above-ground nodes; calculated per plant; plants cultivated in hydroponic system under SD photoperiod; evaluated at pre-tuberization stage (28 DAT) and early tuberization stage (41 DAT), WT and two independent BEL11 RNAi lines: 10 and 12; n= 7-10. Error bars represent the standard deviations. ANOVA-One-Way Analysis of Variance, Dunnett's Two-Sided Multiple-Comparison Test With Control was used for statistical evaluation; asterisks indicate statistically significant differences *** (α=0.001); ** (α =0.01); * (α =0.05); ns (not significant).

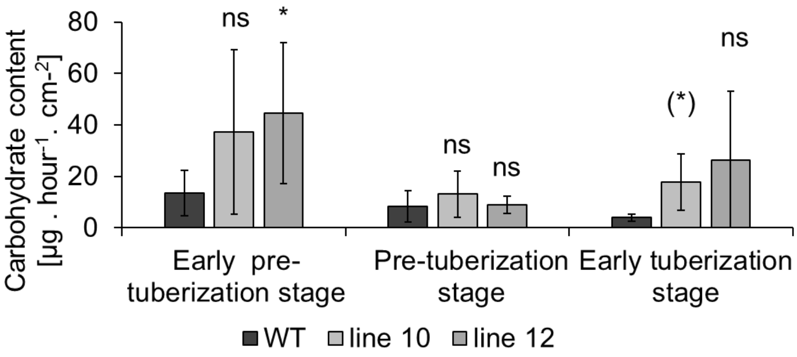

Fig. S12: Carbohydrate content in phloem exudates during tuberization onset: total carbohydrate content related to leaf area; measured in plants cultivated in hydroponic system under SD photoperiod; evaluated at early pre-tuberization stage (21 DAT), pre-tuberization stage (28 DAT) and early tuberization stage (41 DAT), WT and two independent BEL11 RNAi lines: 10 and 12; n= 3-6. Error bars represent the standard deviations. Kruskal-Wallis Z test (Dunn's test) was used for statistical evaluation; asterisks indicate statistically significant differences * (α =0.05); (*) (α = 0.1); ns (not significant).

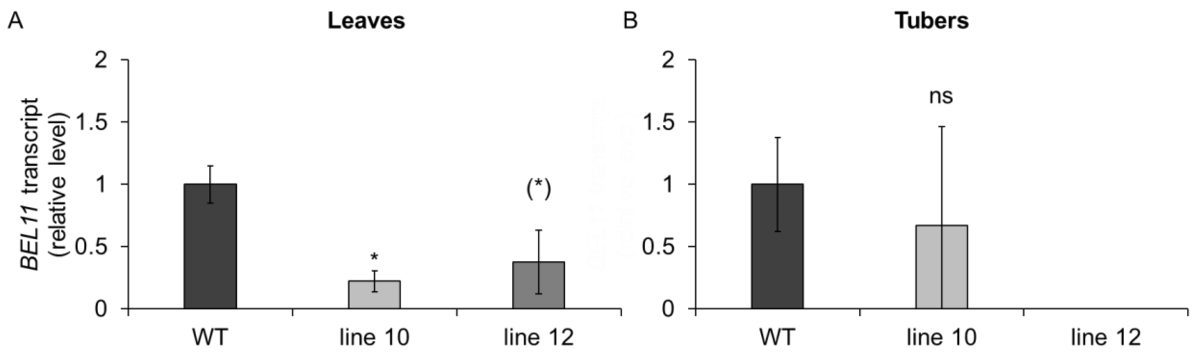

Fig. S13: *BEL11* transcript levels at early tuberization stage: (A) in leaves, (B) in tubers. RNA was isolated from plants cultivated in hydroponic system under SD photoperiod; evaluated 41 DAT (early tuberization stage), WT and two independent BEL11 RNAi lines: 10 and 12. Normalized to the copy number of the reference transcript – polyubiquitin, 3 technical replicates per cDNA dilution (5x). WT level was set to 1 (mean Δ Ct values for WT are provided in Table S2). n= 2-4, error bars represent the standard deviations; ANOVA-One-Way Analysis of Variance, Dunnett's Two-Sided Multiple-Comparison Test With Control was used for statistical evaluation; asterisks indicate statistically significant differences * (α =0.05); (*) (α= 0.1); ns (not significant).
