# Supplementary material for: The role of transcription factor StBEL11 in carbon allocation and tuberization in cultivated potato differs from that known for the model Andean genotype
Source: J Exp Bot. 2025 Dec 24;77(6):1773–88. doi: 10.1093/jxb/eraf551 (PMC13017113; doi:10.1093/jxb/eraf551)
Supplement: eraf551_Supplementary_Data [file eraf551_supplementary_data.zip › jexbot316721-file002.pdf]

Supplementary Table S1: List of primers (5´- 3´)

**Primers for confirmation of transgene presence in BEL11-RNAi lines**		
***CaMV 35S* promoter F**	CTATCCTTCGCAAGACCCTT	
***StBEL11 AS* R**	ACATCATCATCATCAACAACACC	
**Primers for expression analysis (qRT-PCR)**		**Gene ID**
StUBI F	CTTCAAATTTCTCTTTCAAGATGCAG	102599865
StUBI R	AGCCTTTGCTGATCCGGGG	
StBEL11 F	CGCGCTTGGCTTTTCGAGC	102590280
StBEL11 R	TGGCTTCCATAGTCTAACTCTAG	
StBEL5 F	CTTGCCATGCTTGAAGAGGTGG	102577460
StBEL5 R	TGGCTGATCCAATTCCTGCTACTT	
StBEL29 F	CGCAGAGAGGCAAGAAATTCA	102606309
StBEL29 R	CAGCTTGCTCCAACCAGTGTA	
StPOTH1 F	GGAAATTGTGGCGGAGGCA	102577616
StPOTH1 R	GTGCTCCAACCTTTTGGCAAT	
StSP6A F	CAACTTTTACACTCTGATTATGGTG	102577452
StSP6A R	GCTTGTATTTGTAGTTGCTGGGA	

Supplementary Table S2: Overview of RT-qPCR assay parameters for WT under two different cultivation conditions (for details see Methods section): mean Δ Ct (calculated as Ct target transcript in test – Ct reference transcript) ± standard deviation; mean target mRNA amplification efficiency across evaluated organs ± standard deviation; mean reference (*StUBI* mRNA) amplification efficiency across evaluated organs ± standard deviation; approximative relative fold changes of target transcripts in tested organs compared to leaves (calculated as $E_t^{(Ct_t^l - Ct_t^o)} / E_r^{(Ct_r^l - Ct_r^o)}$ using E, mean PCR efficiency; Ct, threshold cycle value; t, target mRNA; r, reference mRNA; l, leaf; o, organ to be compared to leaf).

**Non-tuberizing plants (LD)**					
**Target mRNA**	**Organ**	**Mean Δ Ct**	**Mean target mRNA amplification efficiency**	**Mean reference mRNA amplification efficiency**	**Relative target transcript fold change vs. leaf**
StBEL11	**leaf**	5.657 ± 0.773	2.041 ± 0.074	1.982 ± 0.077	1
	**stolon**	5.824 ± 1.089			0.870
	**roots**	6.237 ± 2.972			0.612
StBEL5	**leaf**	6.723 ± 0.388	1.976 ± 0.038	1.943 ± 0.026	1
	**stolon**	6.831 ± 1.125			0.909
	**root**	5.839 ± 2.602			1.653
StStSP6A	**leaf**	9.841 ± 0.720	1.950 ± 0.076	1.969 ± 0.010	1
	**stolon**	10.016 ± 2.631			0.917
	**root**	10.110 ± 5.596			0.893
StBEL29	**leaf**	9.160 ± 0.977	2.318 ± 0.064	1.961 ± 0.008	1
	**stolon**	9.073 ± 1.428			0.656
StPOTH1	**leaf**	13.22 ± 1.605	1.963 ± 0.082	1.9370 ± 0.023	1
	**stolon**	8.788 ± 0.653			19.786
**Early tuberization stage (SD)**					
**Target mRNA**	**Organ**	**Mean Δ Ct**	**Mean target mRNA amplification efficiency**	**Mean reference mRNA amplification efficiency**	**Relative target transcript fold change vs. leaf**
StBEL11	**leaf**	3.425 ± 0.413	2.041 ± 0.074	1.982 ± 0.077	1
	**tuber**	9.138 ± 0.656			0.024
StSP6A	**leaf**	1.308 ± 2.124	1.950 ± 0.076	1.969 ± 0.010	1
	**tuber**	9.766 ± 0.792			0.003

Supplementary Table S3: The statistical significance of the differences in the levels of individual carbohydrates measured for independent BEL11 RNAi lines: line 1, 10, 12 and 19 versus WT (mean values ± SE) evaluated in source leaves, stolons and roots (see also Fig. 5C-E); n= 3-18, ANOVA-One-Way Analysis of Variance, Dunnett's Two-Sided Multiple-Comparison Test With Control was used for statistical evaluation of data with normal distribution; Kruskal-Wallis Z test (Dunn's test) was used for data not meeting the assumptions of normality; asterisks indicate statistically significant differences (*) (α= 0.1); ns (not significant).

	leaves				stolons				roots			
	sucrose	glucose	fructose + inositol	starch	sucrose	glucose	fructose +inositol	starch	sucrose	glucose	fructose +inositol	starch
**WT**	21,0±2,8	5,0±1,2	11,8±1,4	137,0±14,6	9,3±3,0	104,0±20,5	31,9±15,6	257,3±101,5	7,7±0,8	11,6±1,9	21,5±2, 6	61,0±6,4
**line 1**	ns	ns	ns	ns	ns	ns	ns	ns	ns	ns	ns	ns
**line 10**	ns	(*)	(*)	ns	ns	ns	ns	ns	ns	ns	ns	ns
**line 12**	ns	ns	ns	ns	ns	ns	ns	ns	ns	(*)	ns	(*)
**line 19**	ns	ns	(*)	ns	ns	ns	ns	ns	ns	ns	ns	ns

Supplementary Table S4: The statistical significance of the differences in the levels of individual carbohydrates measured for independent BEL11 RNAi lines: line 1, 10, 12 and 19 versus WT (mean values ± SE) evaluated in source leaves, stolons and roots (see also Supplementary Fig. S7A-C); n= 6-7, ANOVA-One-Way Analysis of Variance, Dunnett's Two-Sided Multiple-Comparison Test With Control was used for statistical evaluation of data with normal distribution; Kruskal-Wallis Z test (Dunn's test) was used for data not meeting the assumptions of normality; asterisks indicate statistically significant differences (*) (α= 0.1); ns (not significant).

	leaves				stolons				roots			
	sucrose	glucose	fructose +inositol	starch	sucrose	glucose	fructose +inositol	starch	sucrose	glucose	fructose +inositol	starch
**WT**	17,3±2,4	38,1±3,9	59,6±5,6	298,2±42,8	11,9±2,5	171,9±17,5	52,2±10,2	593,8±103,0	15,5±2,6	41,0±5,3	69,4±8,1	92,2 ±5,7
**line 1**	ns	ns	ns	ns	**	ns	(*)	ns	ns	ns	ns	ns
**line 10**	*	*	*	ns	ns	ns	ns	ns	ns	ns	ns	*
**line 12**	ns	ns	ns	ns	*	*	ns	ns	ns	ns	ns	ns
**line 19**	ns	ns	ns	**	**	ns	ns	ns	(*)	ns	ns	ns

Supplementary Table S5: The statistical significance of the differences in the levels of individual carbohydrates measured for independent BEL11 RNAi lines: line 10 and 12 versus WT (mean values ± SE) evaluated in source leaves, roots and stolons/tubers at pre-tuberization and early tuberization stage (see also Fig. 7A-C); n= 3-6, ANOVA-One-Way Analysis of Variance, Dunnett's Two-Sided Multiple-Comparison Test With Control was used for statistical evaluation of data with normal distribution; Kruskal-Wallis Z test (Dunn's test) was used for data not meeting the assumptions of normality; asterisks indicate statistically significant differences *** (α= 0.001); ** (α= 0.01); * (α= 0.05); (*) (α= 0.1); ns (not significant); NA – not analyzed.

	**source leaves**							
	**pre-tuberization stage**				**early tuberization stage**			
	**sucrose**	**glucose**	**fructose + inositol**	**starch**	**sucrose**	**glucose**	**fructose + inositol**	**starch**
**WT**	24,4±2,2	3,7±1,2	7,7±1,2	175,0±20,6	28,1±5,1	43,3±6,3	54,7±9,6	138,6±11,9
**line 10**	ns	ns	ns	ns	*	ns	ns	ns
**line 12**	ns	ns	ns	*	ns	**	*	ns
	**roots**							
	**pre-tuberization stage**				**early tuberization stage**			
	**sucrose**	**glucose**	**fructose + inositol**	**starch**	**sucrose**	**glucose**	**fructose + inositol**	**starch**
**WT**	3,1±0,4	4,7±1,3	21,7±4,9	82,5±7,9	9,3±1,5	46,1±6,5	84,4±12,7	138,6±11,9
**line 10**	**	ns	*	*	ns	ns	ns	(*)
**line 12**	***	*	*	(*)	***	***	**	**
	**stolons**				**tubers**			
	**pre-tuberization stage**				**early tuberization stage**			
	**sucrose**	**glucose**	**fructose +inositol**	**starch**	**sucrose**	**glucose**	**fructose +inositol**	**starch**
**WT**	4,7±1,6	108,7±12,5	44,4±10,1	549,6±210,45	11,9±1,7	88,5±18,1	6,8±5,7	469,5±35,5
**line 10**	*	**	ns	*	**	(*)	ns	ns
**line 12**	*	**	*	*	NA	NA	NA	NA
